# Whole-body vibration to prevent intensive care unit-acquired weakness: safety, feasibility, and metabolic response

**DOI:** 10.1186/s13054-016-1576-y

**Published:** 2017-01-09

**Authors:** Tobias Wollersheim, Kurt Haas, Stefan Wolf, Knut Mai, Claudia Spies, Elisabeth Steinhagen-Thiessen, Klaus-D. Wernecke, Joachim Spranger, Steffen Weber-Carstens

**Affiliations:** 1 Department of Anesthesiology and Operative Intensive Care Medicine, Campus Virchow Klinikum and Campus Mitte, Charité—Universitätsmedizin Berlin, Augustenburger Platz 1, Berlin, 13353 Germany; 2 Berlin Institute of Health (BIH), Berlin, 13353 Germany; 3 Department of Neurosurgery, Charité—Universitätsmedizin Berlin, Berlin, 13353 Germany; 4 Department of Endocrinology, Diabetes and Nutrition, Charité—Universitätsmedizin Berlin, Berlin, 10177 Germany; 5 Research Group on Geriatrics, Charité—Universitätsmedizin Berlin, Berlin, 13353 Germany; 6 CRO SOSTANA GmbH Berlin, Berlin, 10318 Germany; 7 DZHK (German Centre for Cardiovascular Research), partner site Charité Berlin, Berlin, 10177 Germany

**Keywords:** Intensive care unit-acquired weakness, Physiotherapy, Whole-body vibration, Mobilization, Muscle wasting, Metabolism

## Abstract

**Background:**

Intensive care unit (ICU)-acquired weakness in critically ill patients is a common and significant complication affecting the course of critical illness. Whole-body vibration is known to be effective muscle training and may be an option in diminishing weakness and muscle wasting. Especially, patients who are immobilized and not available for active physiotherapy may benefit. Until now whole-body vibration was not investigated in mechanically ventilated ICU patients. We investigated the safety, feasibility, and metabolic response of whole-body vibration in critically ill patients.

**Methods:**

We investigated 19 mechanically ventilated, immobilized ICU patients. Passive range of motion was performed prior to whole-body vibration therapy held in the supine position for 15 minutes. Continuous monitoring of vital signs, hemodynamics, and energy metabolism, as well as intermittent blood sampling, took place from the start of baseline measurements up to 1 hour post intervention. We performed comparative longitudinal analysis of the phases before, during, and after intervention.

**Results:**

Vital signs and hemodynamic parameters remained stable with only minor changes resulting from the intervention. No application had to be interrupted. We did not observe any adverse event. Whole-body vibration did not significantly and/or clinically change vital signs and hemodynamics. A significant increase in energy expenditure during whole-body vibration could be observed.

**Conclusions:**

In our study the application of whole-body vibration was safe and feasible. The technique leads to increased energy expenditure. This may offer the chance to treat patients in the ICU with whole-body vibration. Further investigations should focus on the efficacy of whole-body vibration in the prevention of ICU-acquired weakness.

**Trial registration:**

Applicability and Safety of Vibration Therapy in Intensive Care Unit (ICU) Patients. ClinicalTrials.gov NCT01286610. Registered 28 January 2011.

**Electronic supplementary material:**

The online version of this article (doi:10.1186/s13054-016-1576-y) contains supplementary material, which is available to authorized users.

## Background

Muscle wasting and intensive care unit-acquired weakness (ICU-AW) are common complications in ICU patients, leading to longer ICU and hospital stay, higher morbidity and mortality, as well as a poor long-term prognosis [1–3]. Sepsis, multiple organ failure, muscle inactivity, hyperglycemia, as well as the use of corticosteroids and neuromuscular blocking agents were identified as risk factors [1, 4, 5]. ICU-AW diagnosis is often delayed during the ICU stay, usually after a reduction of analgesics and anxiolytics, as the patients first become fully alert. Decreased muscle protein synthesis and increased protein degradation are involved in the pathomechanism, and occur very early during critical illness [6, 7]. Early mobilization of alert patients reduces the length of mechanical ventilation and ICU and hospital stay [8, 9], and leads to better functional independence at hospital discharge [8]. These results only relate to patients who are able to participate in active physiotherapy. Hence follows the idea of closing the gap between onset of critical illness and active muscle training, using external devices during immobilization and sedation phases to evoke muscle contractions [10–13]. During this time course of disease there are further options for intensified passive mobilization by physiotherapists, such as passive cycling or motorized continuous passive motion for different conditions, which we separate from treatment options for active muscle training indicated by patients initiating muscle contraction or from external evoked ones. A series of investigations with electrical muscle stimulation (EMS) in critically ill patients therefore commenced, and while some EMS studies showed promising results [11, 14], others could not [13]. From our own experience we know that application of EMS is time consuming, if feasible at all, and effectiveness is inconsistent [15]. As an alternative, we propose the use of whole-body vibration (WBV) for muscle activation in the ICU. First investigations of human tolerance when exposed to vibration date back to the 1960s [16], and to this day the use of vibration has become more and more interesting in many different approaches and popular in the fitness world. Companies offer devices starting at around €1000. WBV is used as a countermeasure to muscle atrophy and bone loss during the absence of gravity in space, as well as a training option for professional athletes [17, 18] and patients with various underlying diseases [19]. The spinal cord reflex function means that WBV may be suitable for unconscious patients, because muscle contraction occurs at a spinal level and not at a cerebral level [20–22]. There is evidence that prolonged application of WBV helps to maintain muscular mass and strength, increases bone density, improves outcome, and increases glucose metabolism, as shown in healthy volunteers, athletes, older people, or non-ICU patients in the short term [17, 18, 23–30]. These benefits correspond to the needs of critically ill patients and may support ICU patient recovery, although thus far there are no WBV investigations in mechanically ventilated ICU patients. Our aim is to transfer the application of WBV to the ICU.

We hypothesize that the use of WBV in mechanically ventilated ICU patients is safe, feasible, and effective in inducing skeletal muscle activation.

## Methods

### Design

**Fig. 1:**
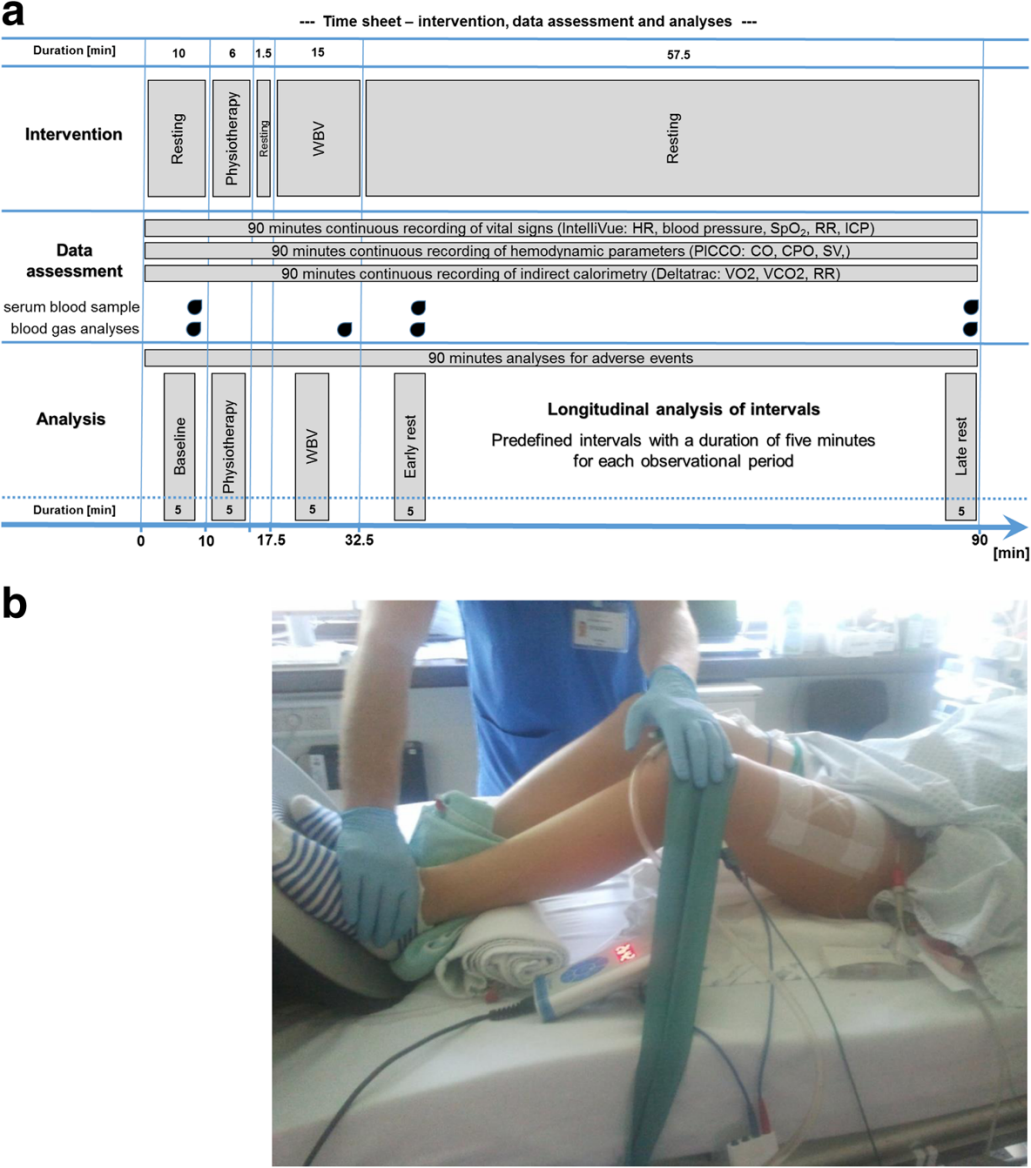
Study protocol and visual presentation of study execution. **a** Visualization of study protocol. Intervention started with 10 minutes of resting, followed by 6 minutes of physiotherapy (passive range of motion of upper and lower extremity). After physiotherapy there was a short resting time, followed by WBV. After WBV, a long resting period took place. Serum blood samples and blood gas analyses were performed at different time points, as shown. Longitudinal analysis of intervals was performed at five different time segments. Analysis was performed at baseline, at physiotherapy, during WBV, and at early and late rest periods. **b** Female patient in a supine position. Vibration device positioned at the end of the bed, with the patient’s feet placed on the middle of the device. An elastic strap is placed around the knee joint to generate pressure on the vibration device. The aim was to flex the knee joint about 20°. The physiotherapist assisted in the stabilization of the lower extremities if necessary. *WBV* whole-body vibration

During a 12-month period, we recruited patients in a mixed ICU and a neurosurgical ICU at a university hospital. In our pilot interventional study, we enrolled critically ill patients who were mechanically ventilated for more than 48 hours with an estimated ICU stay of at least 7 days. Our primary outcome was to show safety and tolerability of WBV by stability of vital parameters (see Additional file 1). Criteria for noninclusion were: lack of informed consent, age < 18 years, preexisting neuromuscular diseases, implanted pacemaker or defibrillator, pregnancy, acute venous thrombosis, unhealed fractures or recently attached implants in body region to be stimulated, recent eye surgery, history of acute herniated discs with acute symptoms, participant in another study, as well as terminal cases. Informed consent was obtained from a legal proxy. The local ethics committee of the Charité (Charité—Universitätsmedizin Berlin, Ethics Commission, Charitéplatz 1, 10117 Berlin, Germany) gave their consent (EA1/017/11). Following a predefined protocol, enrolled patients received passive physiotherapy followed by a single session of WBV. Continuous monitoring of vital signs, hemodynamics, and energy metabolism, as well as intermitted blood sampling (Fig. 1a), took place from the start of baseline measurements up to 1 hour post intervention (for detailed data processing see Additional file 1). The patients were in the supine position during the entire intervention, and no changes in body position took place to avoid any influence on hemodynamic parameters and vital signs. Following baseline measurements, patients were mobilized passively by a physiotherapist for 6 minutes as a warm-up. WBV treatment was then initiated, consisting of a vibration device placed under the patient’s feet, with resistance to the end of the bed. The patient’s hips and knees were flexed at about 20°. An elastic strip provided pressure on the knees, pushing the patient’s feet against the vibration device (Fig. 1b). WBV sessions took 15 minutes, with 9 minutes of clear vibration time. We used two different devices following the manufacturers’ instructions for WBV: one device with synchronous vibration (Promedi, Vibrosphere®, 26 Hz, nine times for 1 minute), and the other with side alternating vibration (Galileo, home-ICU®, 24 Hz, three times for 3 minutes).

Termination criteria for WBV sessions were predefined as follows: heart rate < 40 or > 180 beats per minute; systolic blood pressure < 80 mmHg or > 200 mmHg; mean arterial blood pressure < 60 mmHg or > 120 mmHg; increase in intracerebral pressure > 20 mmHg; SpO_2_ < 88%; or potassium levels < 3.0 mmol/l or > 5.5 mmol/l.

### Data assessment

Data collection was performed using ICM+ software (University of Cambridge) with a recording rate of 50 Hz, where vital signs were monitored using Intellivue (MP30; Phillips) and hemodynamic parameters using PiCCO_2_ (Pulsion Medical Systems, Germany). Indirect calorimetry was performed using Deltatrac (Datex Ohmeda, Finland), and was recorded with Datex Collect with a frequency of one mean per minute. Thermodilution for the PiCCO_2_ system and calibration of all devices took place before each individual session.

We obtained blood gas analyses (BGA) at four time points (Fig. 1a), and measured levels of pO_2_, pCO_2_, pH, sodium, potassium, and blood glucose concentration using a Radiometer ABL 800. Values were used to describe steady-state conditions during the observation, and to observe metabolic response to the intervention. We additionally investigated serum levels of insulin-like growth factor I (IGF-I) and cortisol before and twice after the intervention, because they represent systemic anabolic and catabolic hormones with major influence on the skeletal muscle. Both hormones had been investigated previously within a WBV setting and showed significant changes in healthy controls [31, 32].

### Data analyses

Besides evaluating the continuous recordings to exclude adverse events, we focused our analyses on comparable time intervals for different parts during the observation. Furthermore, we selected similar predefined time intervals of 5-minute recordings, so as to have coherent and comparable longitudinal data for these observations (Fig. 1a). Testing for equivalence of the multiple primary endpoint (heart rate and systolic blood pressure) was performed for the first observations from baseline and WBV therapy as well as for the mean values of the respective phases. Longitudinal analysis examined data in phases from the baseline, physiotherapy, WBV therapy, early resting period (10 minutes after intervention), and late resting period (50 minutes after intervention).

### Statistical analyses

Results are expressed as medians with interquartile range, or as indicated in the legend. After proof of the multiple primary endpoint for equivalence using the confidence interval method and Schuirman’s OST/TOST for means-paired design [33], we analyzed our time-dependent data in a multivariate nonparametric analysis of longitudinal data in a two-factorial design (first factor (dependent): phases, second factor (dependent): time) [34]. Blood analyses over phases were tested by paired Wilcoxon rank tests for depending samples. A two-tailed *p* value < 0.05 was considered statistically significant. All tests of secondary endpoints were conducted in the area of exploratory data analysis. Therefore, no adjustments for multiple testing have been made. Statistical analyses and graphs were performed using R i386 software, version 2.15.3, IBM SPSS statistics, version 22, and SigmaPlot, version 12.

## Results

### Patients

Patients’ baseline characteristics and medical status on the intervention day are presented in Table 1. All 19 study participants completed the intervention. During the entire observation, no patient reached predefined termination criteria or suffered from related adverse events. No endotracheal tube, tracheal cannula, drain, infusion line, ECMO-cannula central venous catheter, or dialyses catheter was dislocated. The application procedure was simple for a physiotherapist and did not influence the clinical routine more than standard physiotherapy. Preparation for WBV is simple and takes less than 3 minutes.

**Table 1:** Characterization of study participants

Study participants, *n*	19
Subgroup Vibrosphere	12
Subgroup Galileo	7
Age, years	54 (52/59)
Gender, male/female	11/7 (57.9%/42.1%)
BMI (kg/m^2^)	28 (24/31)
Diagnosis	
ARDS	9 (47.4%)
Trauma	2 (10.5%)
CNS	8 (42.1%)
Days between ICU admission and intervention	15 (8/18)
Illness severity at ICU admission	
SOFA score	10 (9/13)
SAPS-II	53 (35/78)
Illness severity at intervention day	
SOFA score	9 (6/10)
SAPS-II	48 (38/52)
GCS at intervention day	5 (3/11)
Sedation, RASS at intervention day	–4 (–4/0)
Selective medication during intervention, number of patients received and rate in those	
Norepinephrine, 12 of 19 patients (rate μg/kg/min)	0.100 (0.048/0.140)
Propofol, 3 of 19 patients (rate mg/kg/min)	0.033 (0.031/0.033)
Midazoloam, 3 of 19 patients (rate mg/kg/min)	0.002 (0.001/0.003)
Sufentanil, 10 of 19 patients (rate μg/kg/min)	0.011 (0.003/0.020)
Clonidin, 6 of 19 patients (rate μg/kg/min)	0.013 (0.007/0.014)

Results expressed as medians with interquartile range (median (25th/75th), or as absolute numbers with percentages (%)

*BMI* body mass index, *ARDS* acute respiratory distress syndrome, *CNS* central nervous system, *ICU* intensive care unit, *SOFA* Sequential Organ Failure Assessment, *SAPS-II* Simplified Acute Physiology Score-II, *GCS* Glasgow Coma Scale, *RASS* Richmond Agitation Sedation Scale

### Multiple primary endpoint

Equivalence testing for baseline against WBV therapy of the multiple primary endpoint consisting of heart rate and systolic blood pressure in a means-paired design (equivalence margins: ±20% (mean baseline) each) resulted in significant equivalence (*p* < 0.0001), adjusted for multiple testing, both using first observations and mean values of the respective phases.

### Longitudinal analyses

#### Vital signs

**Fig. 2:**
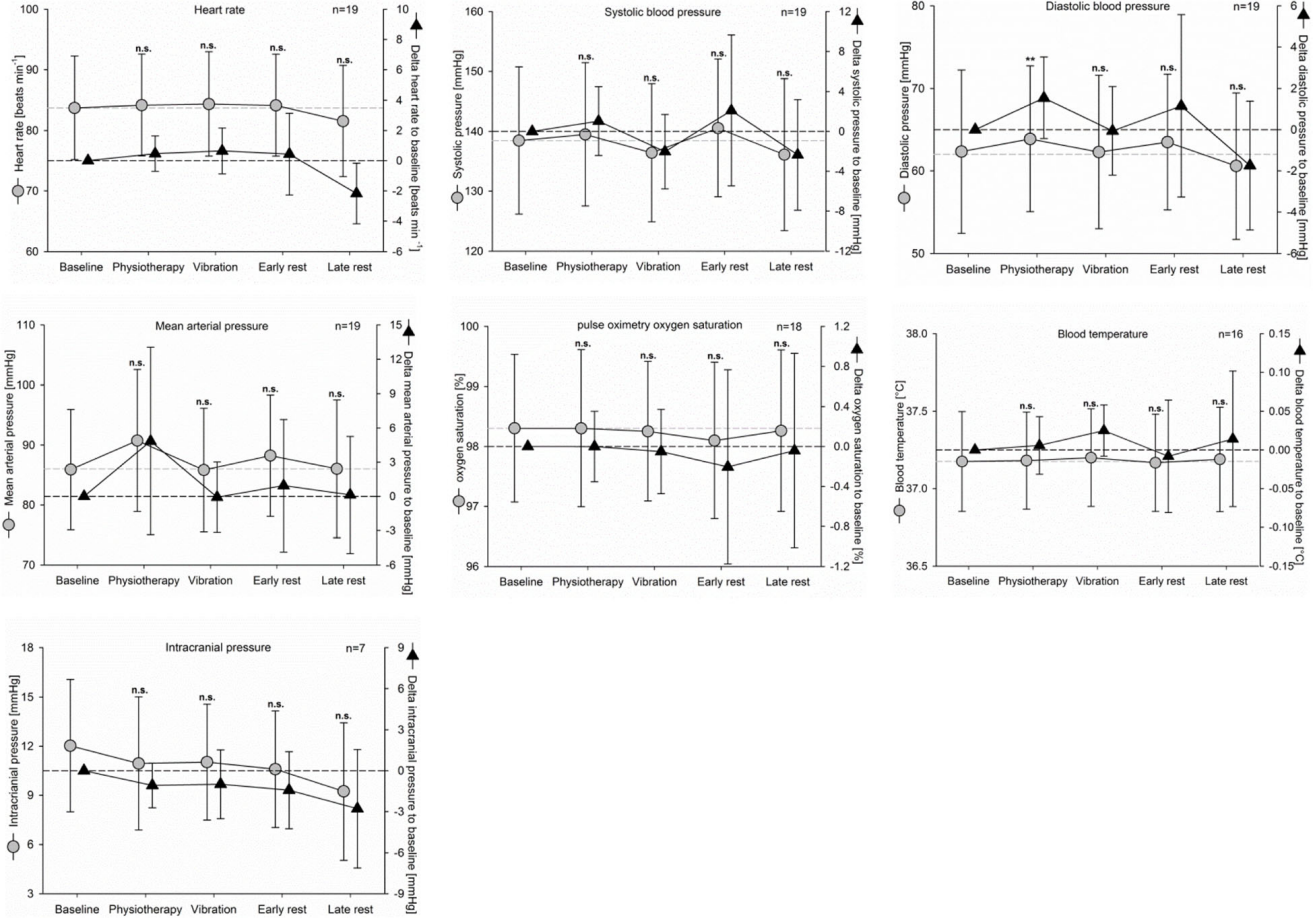
Vital signs for longitudinal observation. *Gray dots and lines*, absolute values; *black triangles and lines*, changes compared with baseline values, mean and 95% CI. *n.s.* not significant to baseline, **p < 0.01

Measurements of vital signs did not significantly change during and after intervention, when compared with baseline (Fig. 2). Minor changes were observed, but were never critical for the patients’ safety. Although the baseline values varied between patients (Fig. 2, gray dots and lines), individual changes were in a small range (Fig. 2, black triangles and lines). Diastolic blood pressure was significantly elevated during the physiotherapy period as compared with baseline (*p* = 0.014), which did not occur during the WBV, early, or late resting periods. Heart rate, mean arterial pressure, systolic blood pressure, and oxygen saturation did not differ significantly from baseline during physiotherapy, WBV, or the resting periods.

#### Intracranial pressure

Out of 19 patients, seven had an extraventricular liquor drain to measure intracranial pressure (Fig. 2). Neither the physiotherapy intervention, in line with previous investigations [35], nor the WBV significantly influenced intracranial pressure levels.

#### Hemodynamics

**Fig. 3:**
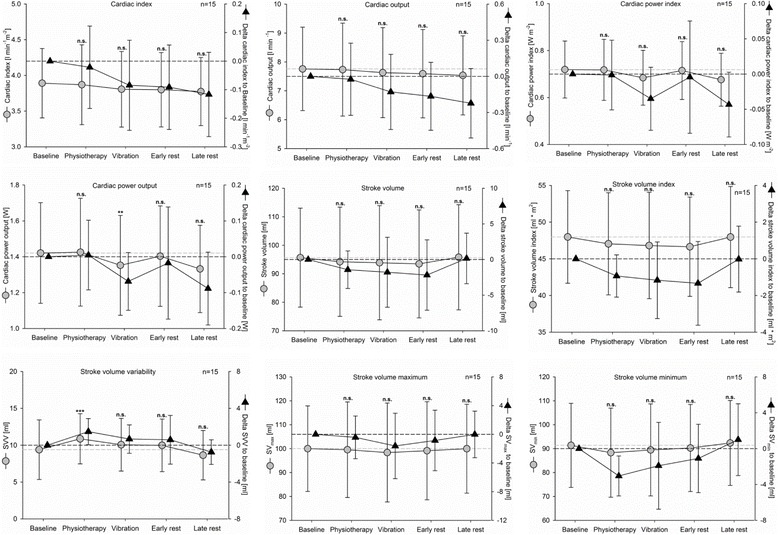
Hemodynamic parameters for longitudinal observation. *Gray dots and lines*, absolute values; *black triangles and lines*, changes compared with baseline values, mean and 95% CI. *n.s.* not significant to baseline, **p < 0.01, ***p < 0.01

Hemodynamic parameters were measured using the PiCCO_2_ Medical-System in a total of 15 patients (Fig. 3). Cardiac output (CO), stroke volume (SV), and stroke volume range (SV minimum, SV maximum) were not significantly influenced by the interventions and remained stable during resting time. Cardiac power output (CPO) showed a significant, but clinically irrelevant decrease during the WBV period compared with baseline (*p* = 0.047), without significant changes in CO and blood pressure. SV variability increased significantly during the physiotherapy period in comparison with the baseline (*p* < 0.001), but was not significantly influenced by WBV or during resting periods when compared with baseline.

#### Energy metabolism

**Fig. 4:**
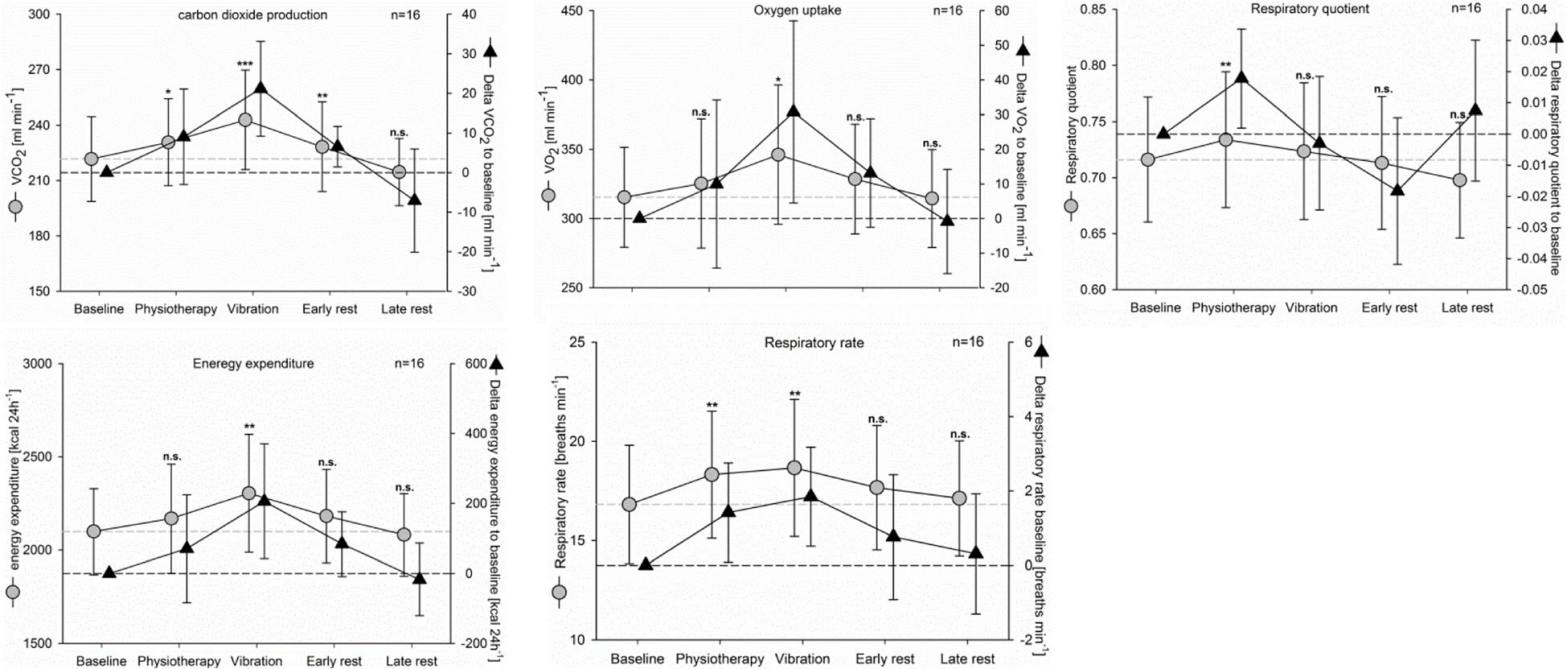
Energy metabolism measurements for longitudinal observation. *Gray dots and lines*, absolute values; *black triangles and lines*, changes compared with baseline values, mean and 95% CI. *n.s.* not significant to baseline, *p < 0.05, **p < 0.01, ***p < 0.01

We measured indirect calorimetry for 16 patients, and found increased energy expenditure (EE) only during WBV (Fig. 4). Comparing the WBV period with the baseline, oxygen uptake levels were significantly increased (*p* = 0.012) and carbon dioxide production was enhanced (*p* < 0.001), showing increased energy expenditure (*p* = 0.007). In contrast, physiotherapy led to increased elimination of carbon dioxide (*p* = 0.041) but not to increased oxygen uptake or increased energy expenditures. During the early and late resting periods, oxygen uptake and energy expenditure did return to baseline values. Carbon dioxide elimination values remained increased during the early resting period (*p* < 0.01), and achieved baseline levels only during the late resting period. Physiotherapy (*p* < 0.01) and WBV (*p* < 0.001) increased the respiratory rate significantly compared with baseline. The respiratory quotient (RQ) increase significant during physiotherapy (*p* = 0.033), which is caused by increased carbon dioxide elimination.

#### Blood analyses

**Fig. 5:**
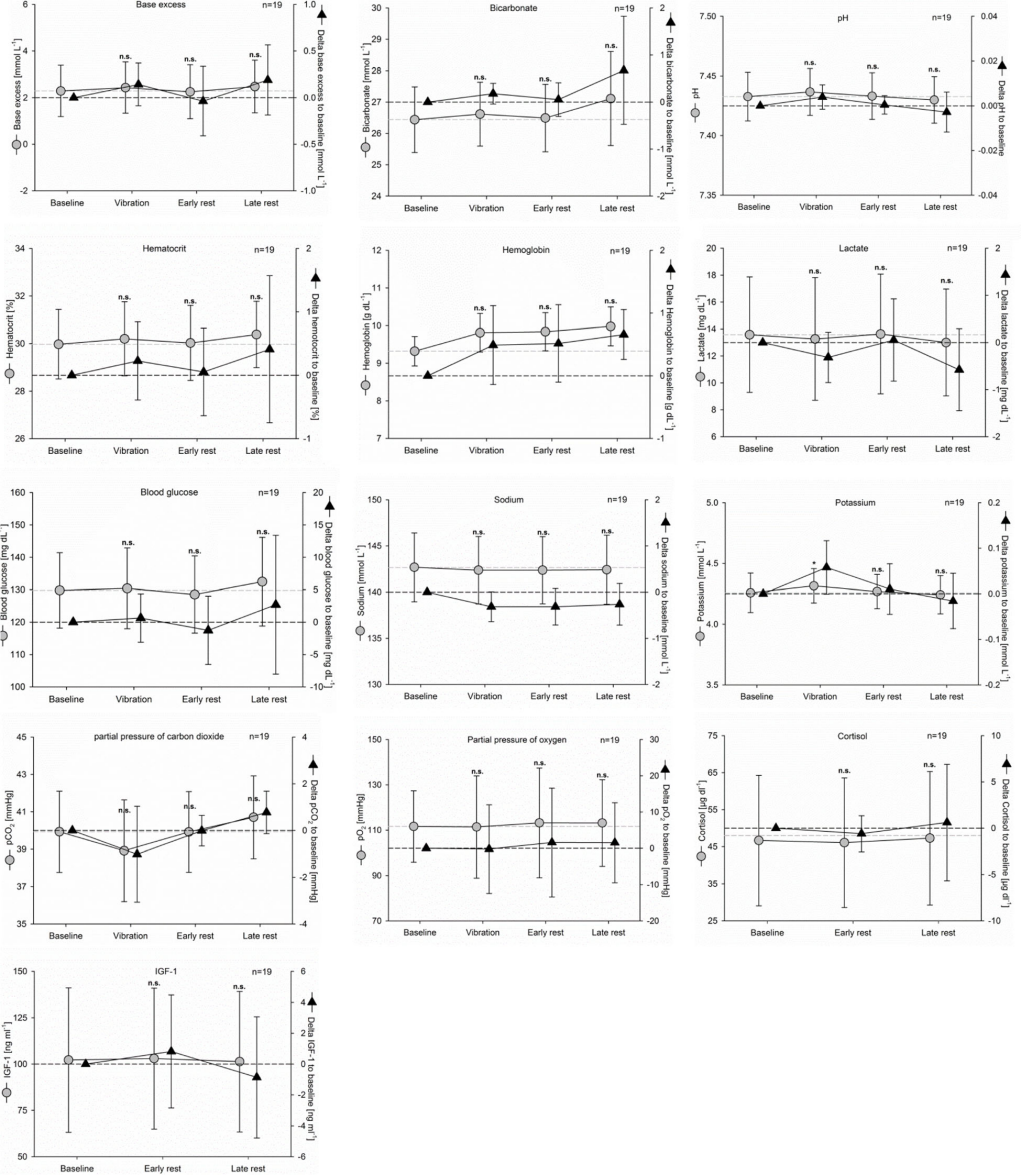
Laboratory blood measurements for longitudinal observation. *Gray dots and lines*, absolute values; *black triangles and lines*, changes compared with baseline values, mean and 95% CI. *n.s.* not significant to baseline, *p < 0.05, **p < 0.01, ***p < 0.01. *IGF-I* insulin-like growth factor I

The BGA (*n* = 19) show a stable ventilation state for the patients, indicated by unchanged pO_2_ and pCO_2_, acid–base state (pH, bicarbonate (HCO_3_^–^), base excess), and oximetry during the entire examination (Fig. 5). WBV was associated with a significant increase of potassium serum levels compared with baseline (*p* = 0.048). This effect was not observed during physiotherapy only. The sodium concentrations within the same blood samples remained unchanged, indicating no errors in the sampling. Furthermore, expected changes for glucose and lactate levels could not be observed. Measuring IGF-1 and cortisol levels resulted in a large range of baseline values, which may have contributed to the fact that no significant changes could be observed.

## Discussion

To the best of our knowledge, this is the first report about safety and feasibility of WBV in critically ill, mechanically ventilated patients. We found that WBV is safely applicable even to critically ill patients in severe condition, as indicated by high SOFA and SAPS-II scores in addition to mechanical ventilation.

Our approach is to induce muscle activation during early critical illness, when patients are unable to participate in active physiotherapy due to sedation or unconsciousness due to neurological reasons. WBV might be an option to evoke muscle activation within a protocol-based physiotherapy and mobilization plan during the course of disease. Additionally, WBV may be a treatment option throughout the ICU stay; that is, may be continued when patients are awake.

The beneficial effect of physiotherapy and early mobilization, which has been shown to be safe and feasible, has been shown in several clinical studies [8, 9, 36, 37]. There are still phases in which patients are not available for active physiotherapy, and these intervals often coincide with intervals of severe illness, acute systemic inflammation, or dependency on norepinephrine for hemodynamic stability. These early periods of critical illness and inflammation are particularly significant in the development of muscle wasting and ICU-AW, as we [6, 14] and others [7] could recently show. Evoked muscle training to avoid immobilization due to EMS can be an option [10–12, 14], but application is labored, often not feasible [15], and in general EMS therapy for ICU patients remains controversial [38]. Alternatively WBV may be able to close the gap between immobilization and active physiotherapy, hypothesizing that frequently applied early muscle activation evoked by WBV may support patient recovery.

WBV represents a strong stimulus to the skeletal muscle, leading to physiological growth adaption in bone and muscle [39, 40]. Clinically, it was shown that WBV improves average velocity, average force, and average power [41] in volunteers and not critically ill patients. The activation on spinal linkage by WBV is evident, as published in a recent investigation showing increased EMG activity on the paretic and nonparetic sides of stroke patients, independent of the intensity of the stimulus [19].

The physiological principal behind WBV is a mechanical stretch and reflex mechanism by the peripheral nerve [20]. Dependent on the frequency of the vibration stimulus, WBV leads to much more than 1000 muscle contractions per minute, leading to increased muscle strength and mass, seen as muscle hypertrophy. This principle of muscle activation agrees with the metabolic findings and expected benefits for ICU patients. Our data show that passive range of motion via physiotherapy increases carbon dioxide elimination, which can be explained by the mobilization of resting blood in the capacity vessels. Absence of active muscle contraction in passive mobilization is reflected by a missing increase in oxygen uptake. In contrast, WBV in critically ill patients increases both carbon dioxide elimination and oxygen uptake in our patients. This has been shown by others in overweight and obese women [42]. The physiotherapist had the subjective impression that, in single cases, patients had an arousal reaction due to the intervention, which was not measurable by RASS scoring but may have an impact on their energy expenditure. We interpret this increased energy turnover as the result of muscular activation. That the increased energy expenditure is caused by actual muscle activation, and not by metabolic dysregulation, is confirmed by steady-state levels for pO2, pCO2, pH, HCO_3_^–^, and base excess. Time delay between intervention and measurement of the indirect calorimetry may occur but is improbable due to the selected time frame and no significant changes over time within each phase (see Additional file 1). Serum potassium levels were significantly increased only during WBV, probably due to muscle contraction, and unchanged serum sodium levels underline our interpretation.

Besides the mechanical stretch and reflex mechanism by the peripheral nerve caused by the vibration stimuli, there is evidence for an additional, direct impact on different tissues. This could be demonstrated by molecular findings showing beneficial effects of vibration in vivo and in vitro on separated stem cells, myoblasts, and muscle tissue [40, 43, 44]. Ceccarelli et al. [40] showed an increased synthesis and decreased activation of the ubiquitin–proteasome pathway with myostatin and Atrogin-1 suppression in vitro due to vibration. These findings imply that vibration could have a significant impact on maintaining muscle in ICU patients because decreased myosin synthesis and increased myosin degradation is an established mechanism in the development of ICU-AW [6].

Repetitive WBV was shown to have a positive effect on glucose metabolism in type II diabetes patients [27, 28]. We showed recently that EMS has an impact on maintaining muscular mass by improving glucose metabolism in the critically ill [14]. Future studies could investigate whether a similarly positive effect can be achieved by WBV.

We also did not find a serum lactate elevation, which might be expected during extensive muscle training. Thus, WBV does not result in substantial anaerobic muscle activity, which would presumably not be favorable in critically ill patients. Small changes were probably not measurable in an intervention of this scale. Small changes would also explain why we could not find any significant changes in the hormonal regulation of IGF-1 and cortisol, which were shown earlier for both hormones [31, 32].

This pilot study was limited to investigate safety, feasibility, and metabolic response of WBV in critically ill patients, focusing on hemodynamic stability. Thus it was outside the scope of the study to evaluate aspects such as patient comfort, staff workload, and staff acceptance. Further investigations are also needed to assess the most favorable type, intensity, frequency, and duration of WBV in ICU treatment. For the first time in critically ill patients, we could show a safe feasibility of WBV, as well as measure indicators for muscle activation and induced metabolism. These results could be further improved by measuring the muscle activity by electromyography. The next step would be an investigation to determine whether WBV could improve short-term and long-term outcome for ICU patients, by prevention or treatment, as already shown for non-ICU patients.

## Conclusion

We conclude—under consideration of the absolute contraindications—that the application of WBV is safe and feasible in critically ill patients. Our results support the principle that WBV stimulates muscle and improves muscle metabolism, and therefore may have the potential to prevent and/or treat muscle weakness in critically ill patients. Further clinical trials are needed to investigate beneficial effects.

## Additional file

Additional file 1: Supplement Extended methods: From study execution to the results. Statistical methods. Outcome parameters. Definition of passive and active physiotherapy. Extened results: Detailed results of two-factorial design. Results of three-factorial design. Subgroup analyses between differnet WBV devices. (PDF 2922 kb)

## Abbreviations

BGA: Blood gas analyses
CO: Cardiac output
CPO: Cardiac power output
EE: Energy expenditure
EMG: Electromyography
EMS: Electrical muscle stimulation
ICU-AW: Intensive care unit-acquired weakness
IGF-I: Insulin-like growth factor I
pCO2: Partial pressure of carbon dioxide
pO2: Partial pressure of oxygen
SAPS-II: Simplified Acute Physiology Score-II
SOFA: Sepsis-related Organ Failure Assessment
SV: Stroke volume
WBV: whole-body vibration
