# Supplementary material for: Whole-body vibration to prevent intensive care unit-acquired weakness: safety, feasibility, and metabolic response
Source: Crit Care. 2017 Jan 9;21:9. doi: 10.1186/s13054-016-1576-y (PMC5220605; doi:10.1186/s13054-016-1576-y)
Supplement: Additional file 1: — Supplement Extended methods: From study execution to the results. Statistical methods. Outcome parameters. Definition of passive and active physiotherapy. Extened results: Detailed results of two-factorial design. Results of three-factorial design. Subgroup analyses between differnet WBV devices. (PDF 2922 kb) [file 13054_2016_1576_MOESM1_ESM.pdf]

Supplement to: Whole-body-vibration to prevent intensive care unit acquired weakness (ICU-AW): safety, feasibility and metabolic response

Tobias Wollersheim[1,2]*, Kurt Haas[1]*, Stefan Wolf[3], Knut Mai[2,4], Claudia Spies[1], Elisabeth Steinhagen-Thiessen[5], Joachim Spranger[2,4,6], Klaus-D. Wernecke[1,7], Steffen Weber-Carstens[1,2]

* contributing equally

Tobias.wollersheim@charite.de; kurt.haas@st.ovgu.de; stefan.wolf@charite.de; knut.mai@charite.de; Claudia.spies@charite.de; elisabeth.steinhagen-thiessen@charite.de, joachim.spranger@charite.de; wernecke@sostana.de; steffen.weber-carstens@charite.de;

1 Charité - Universitätsmedizin Berlin, Campus Virchow & Campus Mitte, Anesthesiology and Operative Intensive Care Medicine, 13353 Berlin, Germany
2 Berlin Institute of Health (BIH), 13353 Berlin, Germany
3 Charité – Universitätsmedizin Berlin, Department of Neurosurgery, 13353 Berlin, Germany
4 Charité - Universitätsmedizin Berlin, Department of Endocrinology, Diabetes and Nutrition, 10177 Berlin, Germany
5 Charité – Universitätsmedizin Berlin, Research Group on Geriatrics, 13353 Berlin, Germany
6 DZHK (German Centre for Cardiovascular Research), partner site Charité Berlin – 10177 Berlin, Germany
7 CRO SOSTANA GmbH Berlin – 10318 Berlin, Germany

Corresponding author:

PD Dr. med. Steffen Weber-Carstens, Dept. of Anesthesiology and Operative Intensive Care Medicine, Campus Virchow Klinikum and Campus Mitte, Charité - Universitätsmedizin Berlin, Augustenburger Platz 1, 13353 Berlin, Germany.
E-mail: steffen.weber-carstens@charite.de
phone: +49 30 450 651055; fax: +49 30 450 551909

EXTENDED METHODS:

From study execution to the results

Intervention: As shown in figure 1, we performed a series of interventions for each patient.

1. There was a 10-minute resting period where we assessed baseline values prior to the intervention.
2. A physiotherapist moved the patient according to their passive range of motion as a warm up before the start of WBV. This physiotherapeutic session lasted 6 minutes.
    a. A small 90-second pause, without any special intervention, followed the physiotherapeutic session. This time was required to position the vibration device and prepare to start the WBV.
3. We performed the WBV itself. The duration of the WBV phase was 15 minutes. Within these 15 minutes, there was a net vibration time of 9 minutes, intercalated with small resting times totaling 6 minutes. Since WBV is such an intensive stimulus, and according to the manufacture's guidelines, we performed this trial with "interval" stimulation.
4. Patients were monitored for nearly one hour following WBV.

Data assessment: Due to technical reasons, we had different frequencies recording our data.

1. Vital signs were recorded online with a frequency of 50 hertz for a total time of 90 minutes. The Phillips Intellivue monitoring and the Pulsion PICCO system generated 3,000 values per minute, which amounted to 270,000 values for the 90 minutes recording, for each parameter.
2. We used the Deltatrac to perform indirect calorimetry for the same 90 minutes, calculating a mean value per minute that were automatically recorded by the Datex software. This resulted in 90 values for 90 minutes of indirect calorimetry.
3. Blood samples were collected intermittently during the recording time. A total of four BGAs were obtained per patient, each within the last minute of the following phases: baseline, WBV, early resting time, and late resting time. Additionlly, three blood samples for laboratory analyses were obtained, each within the last minute of the following phases: baseline, early resting time, and late resting time.

Analysis: We analyzed the large data sheets and combined the data of different devices into useful data sets.

1. During collection, as well as retrospectively, we screened the full data sets to detect any adverse events, or parameters reaching the abort criteria.
2. For each phase (baseline, physiotherapy, WBV, early rest, late rest), we used predefined intervals with a duration of five minutes. These 5 minutes for each phase serve two purposes. On the one hand, they reflect the different phases of intervention or observation, and on the other hand, they are similar in duration and number of values, which is essential for longitudinal analyses, as recommended by statisticians.
    - The five minute interval was predefined by timescale and similar for all patients, so that the selected five minutes reflect equal timeslots within the intervention series. This allows us to compare the different phases to each other under consideration of their different duration.

- In order to utilize the data without risk of over interpretation, we calculated a mean value for every ten seconds of the high frequency data (50 hertz) for each parameter and for each patient.

For example: The recorded 500 heartrate values (50 hertz recording) obtained in ten seconds were used to calculate a mean heartrate value for each time interval of ten seconds. So out of the 15,000 values per 5-minute interval, we calculated 30 mean values every 10 seconds. These 30 intervals were used to perform the longitudinal data analysis. For all parameters measured by indirect calorimetry, which were already recorded as mean per minute, we used the 5 values for 5 minutes to perform the analyses.

Statistical analyses: Investigation of longitudinal dependent samples was performed according to the algorithm published by Brunner. Within the statistical analysis, this model uses 30 values for 5 minute baseline, 30 values for 5 minute physiotherapeutic phase, 30 values for 5 minute WBV phase, 30 values for 5 minute early resting time, and 30 values for 5 minute late resting time, for all parameters measured by 50 hertz. Similarity, for the indirect calorimetry, 5 values for 5 minute baseline, 5 values for 5 minute physiotherapy phase, 5 values for 5 minute WBV phase, 5 values for 5 minute early resting time, and 5 values for 5 minute late resting time, for all parameters measured by indirect calorimetry were used. In contrast to this longitudinal, repeated, and sequential approach, the blood samples were tested by Wilcoxon range test due to sequential, but single (non-repeated), measurements for each parameter within the individual phases.

Plots: To clearly present the movement through the intervention series and to provide a simplified view of the changes caused by WBV and recovery, we calculated one mean value per 5 minute interval per patient, showing these values in the figures of the main manuscript. The given significances were calculated as described above.

Statistical methods:

Three-factorial-designs in longitudinal analyses:

Including different vibration devices into the evaluation, the above defined multivariate nonparametric analysis of longitudinal data in the two-factorial design has been extended by a third (independent) factor “vibration device”.

Two-factorial designs in longitudinal subgroup analyses:

The two-factorial design from the main manuscript was used in subgroup analyses for every device [Galileo (GAL-Group) and Vibroshpere (VIB-group)] separately, comparing selected phases.

Blood parameters were analyzed over the phases using a nonparametric test (Paired Wilcoxon Signed Rank Test).

A two-tailed p-value < 0.05 was considered statistically significant. All tests were conducted in the area of exploratory data analysis. Therefore, no adjustments for multiple testing have been made.

Descriptive data is presented as mean with standard deviation (SD).

Relative effect p_{is}:

The relative effect of a clinical parameter for a certain group of treatments does not represent the measured values of that parameter, but presents (in a scale between 0 and 1) the treatment effect of the regarded group, relative to all other groups, therefore to a “mean “ treatment effect. Accordingly, a greater relative effect of one group compared to another group means a higher treatment effect of the regarded group and therefore greater values of the corresponding parameter.

Outcome Parameters:

The following outcome parameters were evaluated in the statistical analyses:

Multiple Primary Outcome Measure:

- *Vital parameters “systolic blood pressure” and “heart rate” as measures of hemodynamic stability to show safety and tolerability in the sense of no change (equivalence)[Time Frame: Continuous record for 90 minutes - within 4 blood samples - vibration therapy compared to baseline]*
  [Designated as safety issue: Yes]

Secondary Outcome Measures:

- *Changes in heart rate, arterial blood pressure, saturation of peripheral pulse oximetry, intracranial pressure, mechanical ventilation parameters, electrolytes, blood sugar and lactate levels, blood pH, PICCO-parameters, body temperature as well as pO2 und pCO2 partial pressure.”*

Definition of passive and active physiotherapy:

<u>Passive physiotherapy:</u> Any physiotherapeutic treatment, which does not depend on patients alertness, cooperation or the ability to contract muscles voluntarily. Techniques can be used in the sedated patient.

Typical and often used techniques are passive movements of the limbs to maintain or improve range of motion of the joint or reduce pain, the application of heat to reduce pain, cryotherapy to reduce inflammation, chest therapy (including body positioning, turning) leading to an easier breathing. Massage techniques are also helpful to reduce muscular tonus.

<u>Active physiotherapy:</u> Any physiotherapeutic treatment, which needs the participation and cooperation of the patient. The patient is able to contract the skeletal muscles voluntarily, even if contraction is poor

Active physiotherapy techniques involve the movement of the limbs to maintain and improve muscle strength and range of motion. Stretching techniques can be considered to reduce muscular tonus and improve range of motion. Walking on the treadmill or cycling can enhance stamina. Chest therapy includes relaxing positioning and turning in bed. “

Extended Results

Two-factorial design (1st factor (dependent): phases, 2nd factor (dependent): Time)

Heart Rate

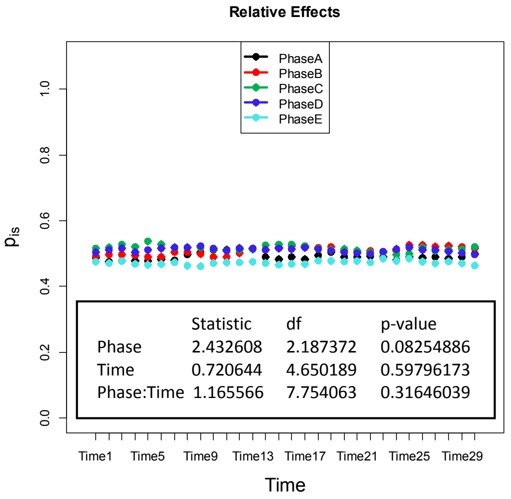

- Phase A – Baseline
- Phase B – Physiotherapy
- Phase C – WBV (whole-body-vibration)
- Phase D – Early Rest
- Phase E – Late Rest

Detailed data presentation and statistical results for longitudinal data assessment. Algorithm of Brunner used for calculation. Time indicates 30 sequential mean values (6 per min) during the compared phases.

Baseline vs. Physiotherapy

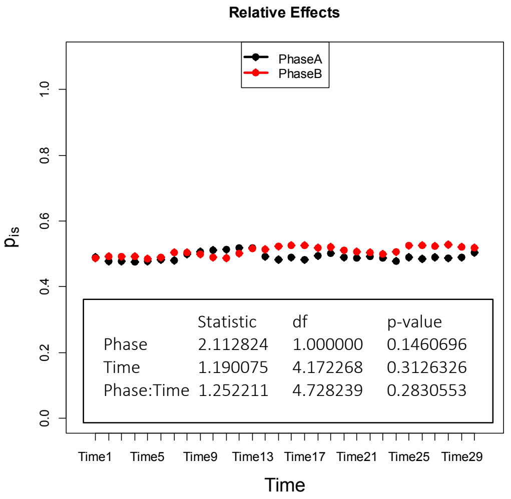

Baseline vs. WBV

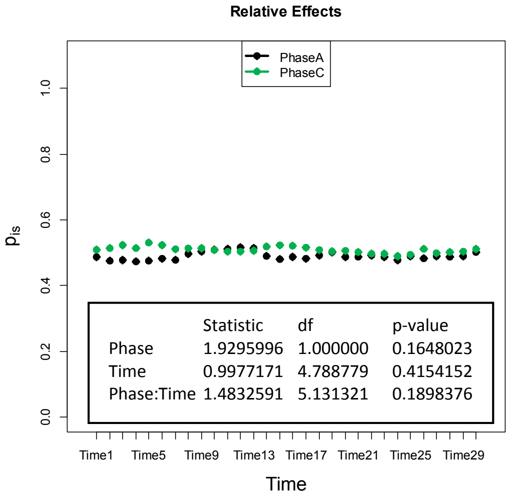

Baseline vs. Early Rest

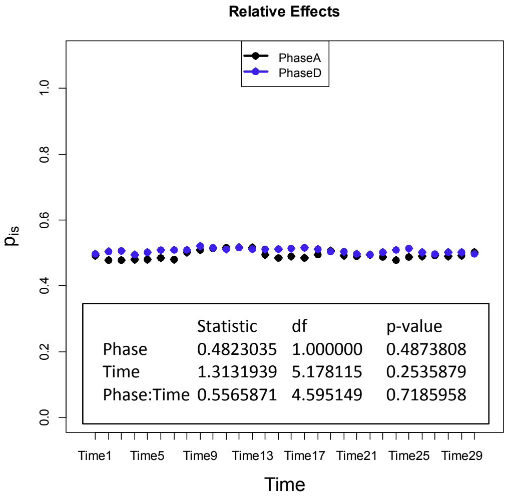

Baseline vs. Late Rest

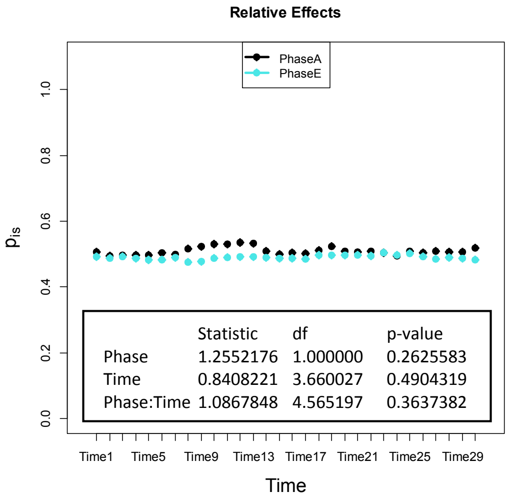

Systolic Blood Pressure

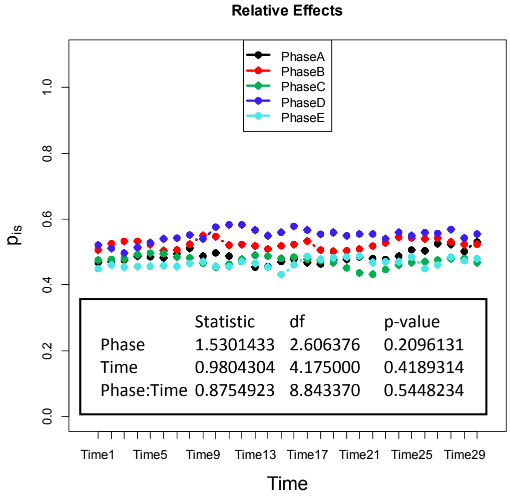

	Statistic	df	p-value
Phase	1.5301433	2.606376	0.2096131
Time	0.9804304	4.175000	0.4189314
Phase:Time	0.8754923	8.843370	0.5448234

- Phase A – Baseline
- Phase B – Physiotherapy
- Phase C – WBV (whole-body-vibration)
- Phase D – Early Rest
- Phase E – Late Rest

Detailed data presentation and statistical results for longitudinal data assessment. Algorithm of Brunner used for calculation. Time indicates 30 sequential mean values (6 per min) during the compared phases.

Baseline vs. Physiotherapy

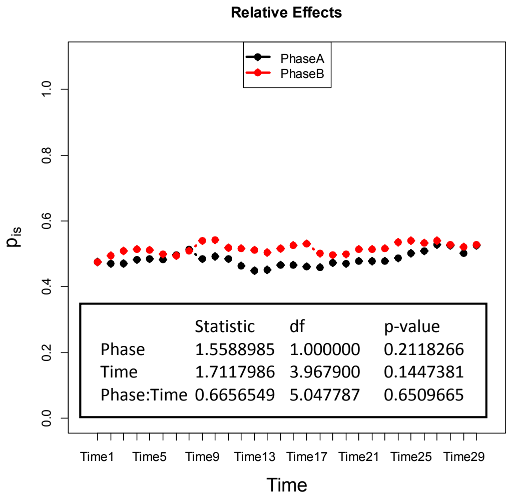

	Statistic	df	p-value
Phase	1.5588985	1.000000	0.2118266
Time	1.7117986	3.967900	0.1447381
Phase:Time	0.6656549	5.047787	0.6509665

Baseline vs. Vibration

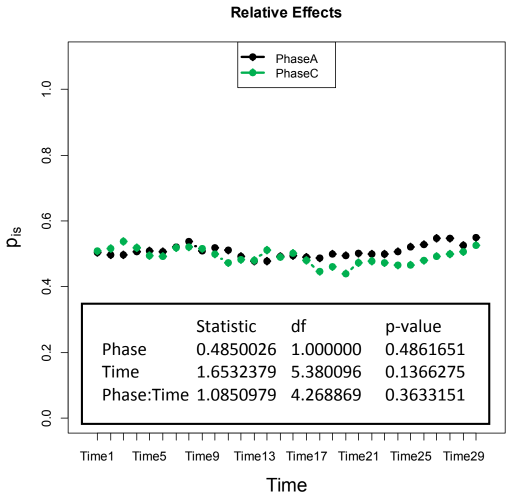

	Statistic	df	p-value
Phase	0.4850026	1.000000	0.4861651
Time	1.6532379	5.380096	0.1366275
Phase:Time	1.0850979	4.268869	0.3633151

Baseline vs. Early Rest

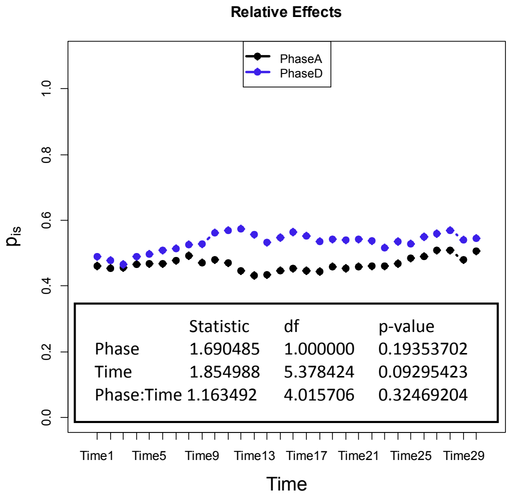

	Statistic	df	p-value
Phase	1.690485	1.000000	0.19353702
Time	1.854988	5.378424	0.09295423
Phase:Time	1.163492	4.015706	0.32469204

Baseline vs. Late Rest

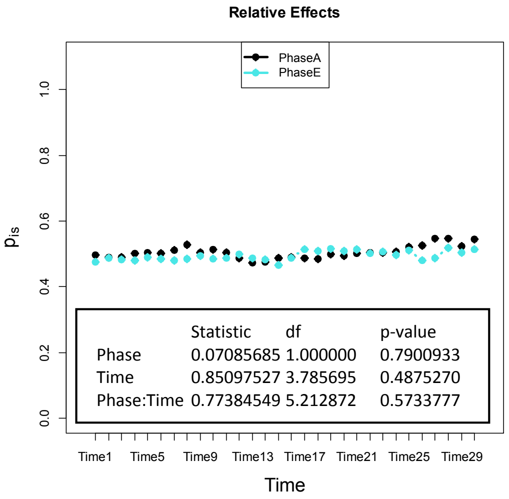

	Statistic	df	p-value
Phase	0.07085685	1.000000	0.7900933
Time	0.85097527	3.785695	0.4875270
Phase:Time	0.77384549	5.212872	0.5733777

Diastolic Blood Pressure

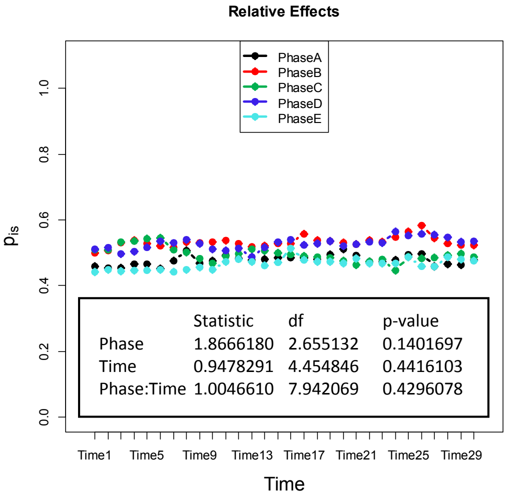

	Statistic	df	p-value
Phase	1.8666180	2.655132	0.1401697
Time	0.9478291	4.454846	0.4416103
Phase:Time	1.0046610	7.942069	0.4296078

- Phase A – Baseline
- Phase B – Physiotherapy
- Phase C – WBV (whole-body-vibration)
- Phase D – Early Rest
- Phase E – Late Rest

Detailed data presentation and statistical results for longitudinal data assessment. Algorithm of Brunner used for calculation. Time indicates 30 sequential mean values (6 per min) during the compared phases.

Baseline vs. Physiotherapy

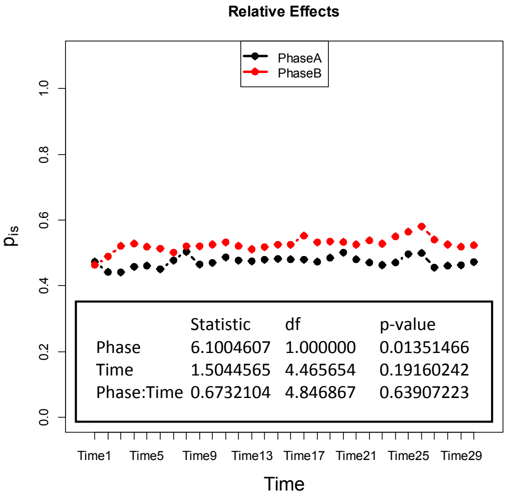

	Statistic	df	p-value
Phase	6.1004607	1.000000	0.01351466
Time	1.5044565	4.465654	0.19160242
Phase:Time	0.6732104	4.846867	0.63907223

Baseline vs. Vibration

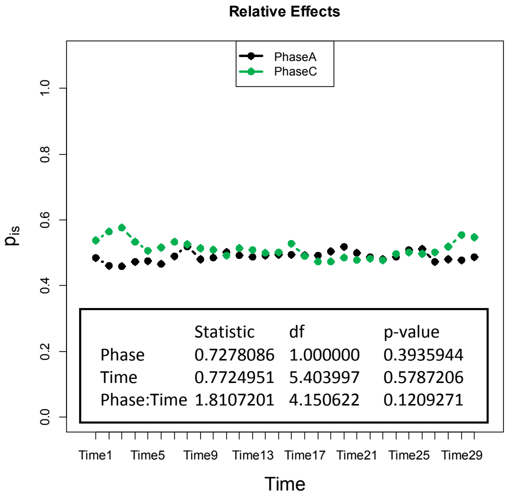

	Statistic	df	p-value
Phase	0.7278086	1.000000	0.3935944
Time	0.7724951	5.403997	0.5787206
Phase:Time	1.8107201	4.150622	0.1209271

Baseline vs. Early Rest

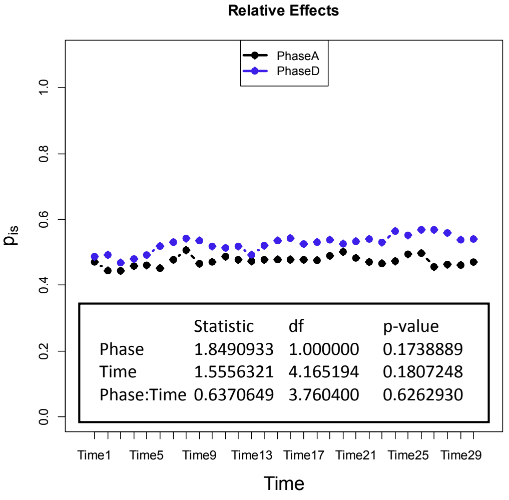

	Statistic	df	p-value
Phase	1.8490933	1.000000	0.1738889
Time	1.5556321	4.165194	0.1807248
Phase:Time	0.6370649	3.760400	0.6262930

Baseline vs. Late rest

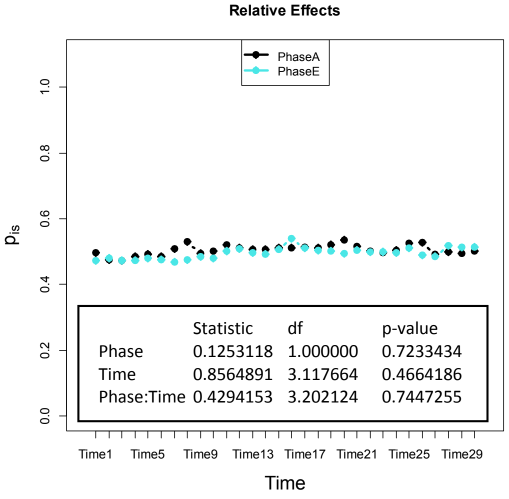

	Statistic	df	p-value
Phase	0.1253118	1.000000	0.7233434
Time	0.8564891	3.117664	0.4664186
Phase:Time	0.4294153	3.202124	0.7447255

Intracranial Pressure

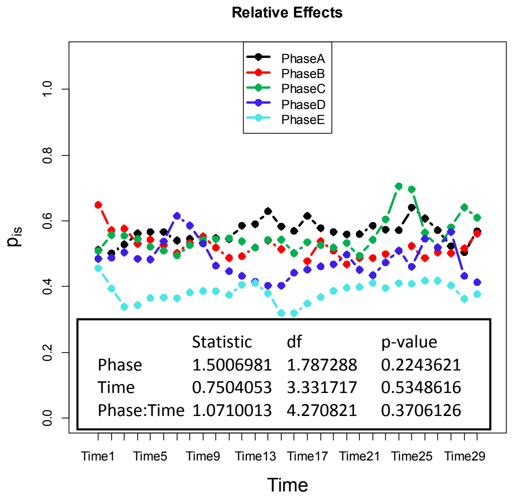

● Phase A – Baseline
● Phase B – Physiotherapy
● Phase C – WBV (whole-body-vibration)
● Phase D – Early Rest
● Phase E – Late Rest

Detailed data presentation and statistical results for longitudinal data assessment. Algorithm of Brunner used for calculation. Time indicates 30 sequential mean values (6 per min) during the compared phases.

Baseline vs. Physiotherapy

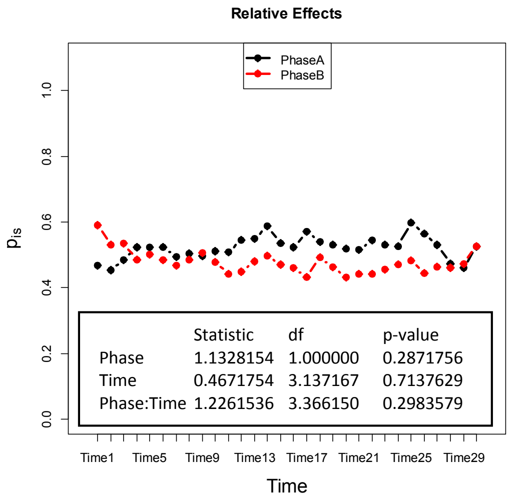

Baseline vs. Vibration

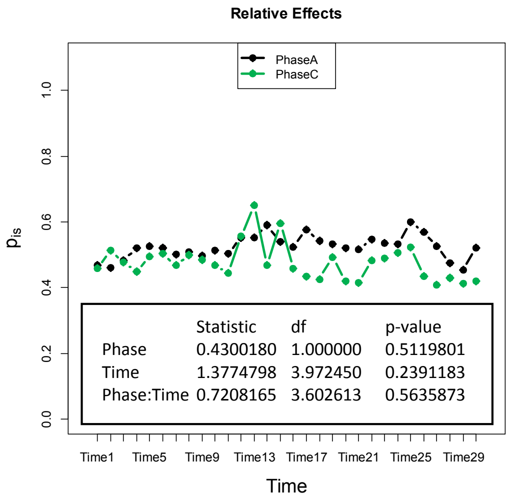

Baseline vs. Early Rest

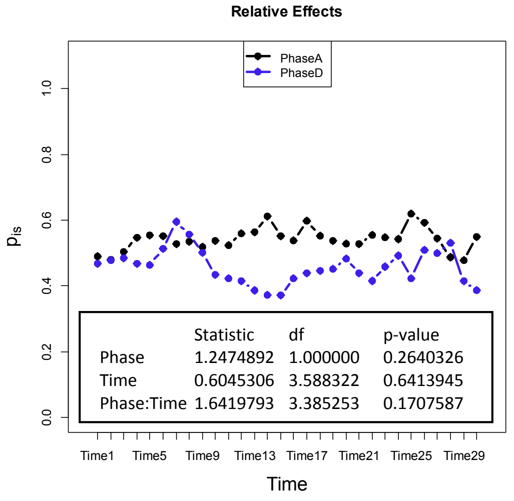

Baseline vs. Late Rest

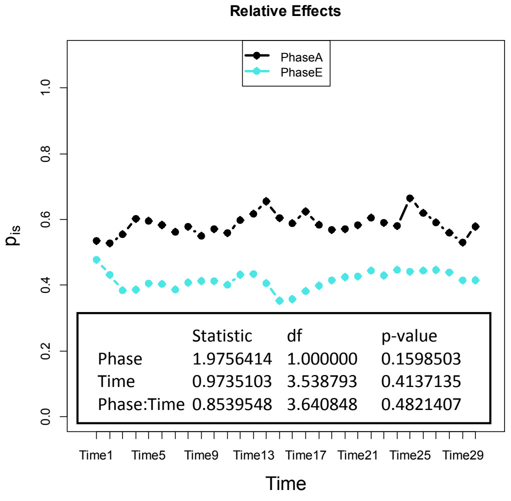

Peripheral Oxygen Saturation (SpO_2)

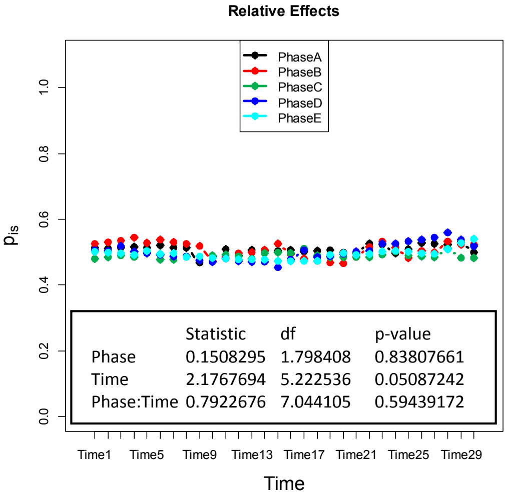

	Statistic	df	p-value
Phase	0.1508295	1.798408	0.83807661
Time	2.1767694	5.222536	0.05087242
Phase:Time	0.7922676	7.044105	0.59439172

- Phase A – Baseline
- Phase B – Physiotherapy
- Phase C – WBV (whole-body-vibration)
- Phase D – Early Rest
- Phase E – Late Rest

Detailed data presentation and statistical results for longitudinal data assessment. Algorithm of Brunner used for calculation. Time indicates 30 sequential mean values (6 per min) during the compared phases.

Baseline vs. Physiotherapy

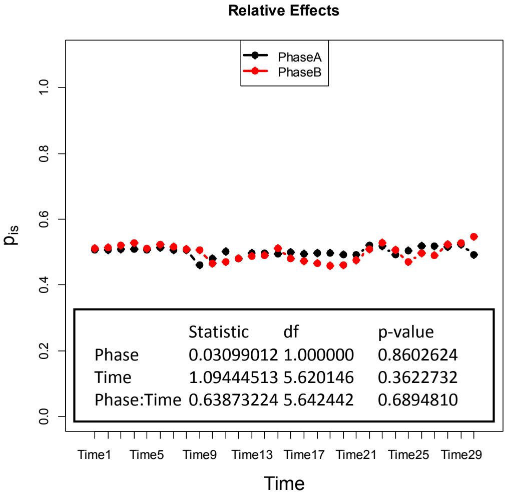

	Statistic	df	p-value
Phase	0.03099012	1.000000	0.8602624
Time	1.09444513	5.620146	0.3622732
Phase:Time	0.63873224	5.642442	0.6894810

Baseline vs. Vibration

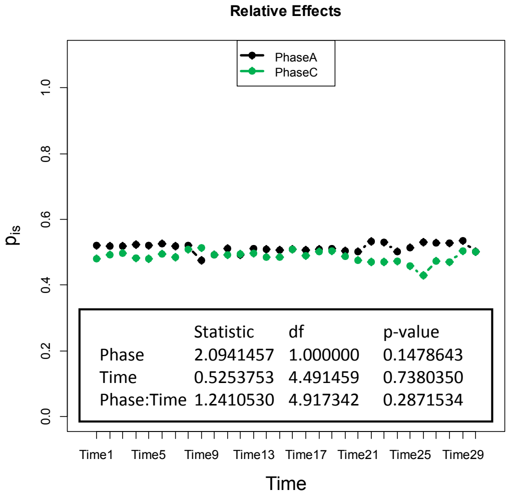

	Statistic	df	p-value
Phase	2.0941457	1.000000	0.1478643
Time	0.5253753	4.491459	0.7380350
Phase:Time	1.2410530	4.917342	0.2871534

Baseline vs. Early Rest

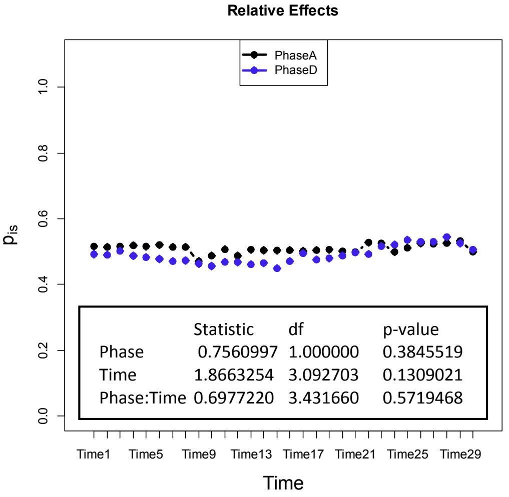

	Statistic	df	p-value
Phase	0.7560997	1.000000	0.3845519
Time	1.8663254	3.092703	0.1309021
Phase:Time	0.6977220	3.431660	0.5719468

Baseline vs. Late Rest

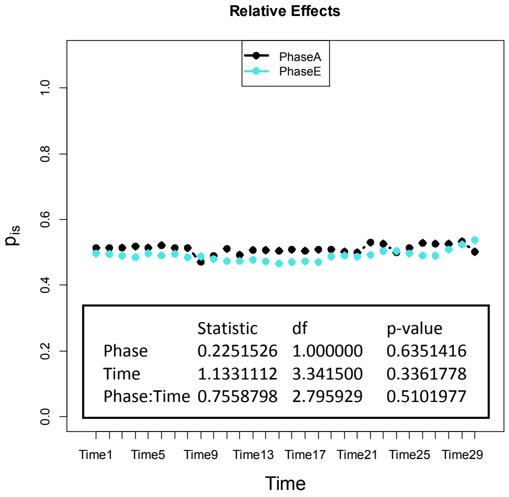

	Statistic	df	p-value
Phase	0.2251526	1.000000	0.6351416
Time	1.1331112	3.341500	0.3361778
Phase:Time	0.7558798	2.795929	0.5101977

Mean Arterial Pressure

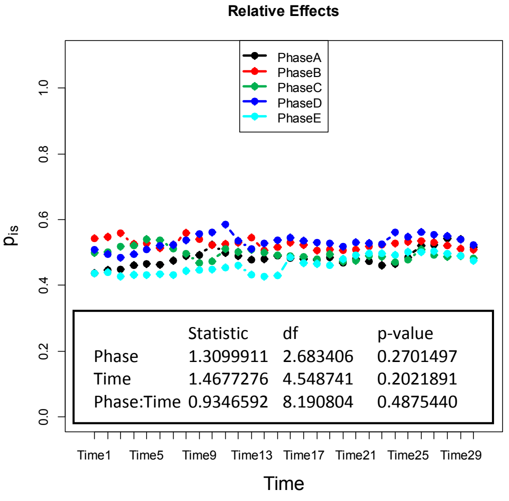

- Phase A – Baseline
- Phase B – Physiotherapy
- Phase C – WBV (whole-body-vibration)
- Phase D – Early Rest
- Phase E – Late Rest

Detailed data presentation and statistical results for longitudinal data assessment. Algorithm of Brunner used for calculation. Time indicates 30 sequential mean values (6 per min) during the compared phases.

Baseline vs. Physiotherapy

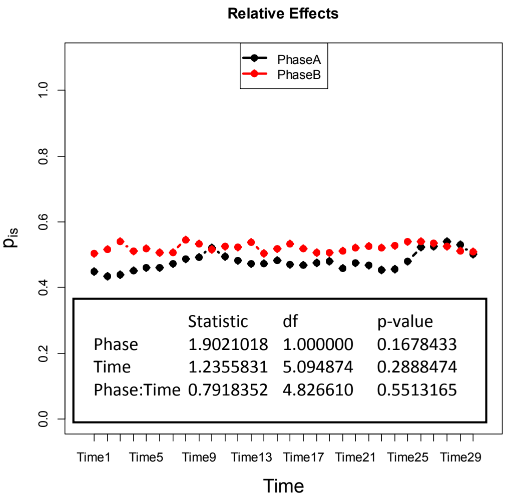

Baseline vs. Vibration

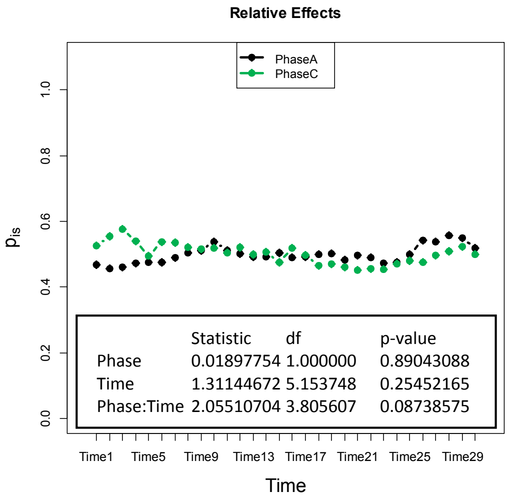

Baseline vs. Early Rest

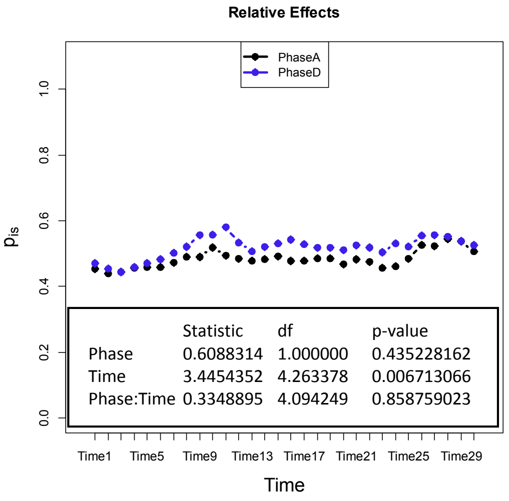

Baseline vs. Late Rest

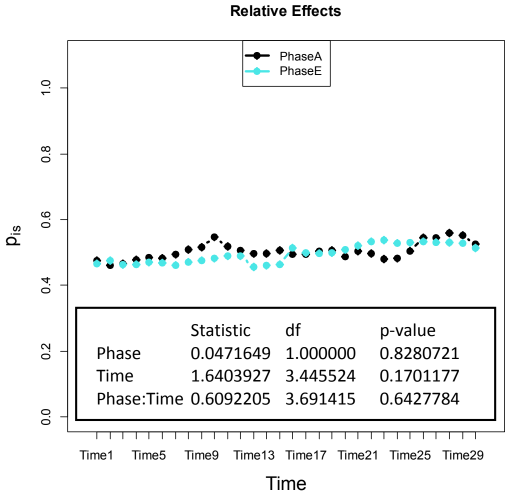

Hemodynamic Parameters

Two-factorial design (1st factor (dependent): phases, 2nd factor (dependent): Time)

Cardiac Power Index (CPI)

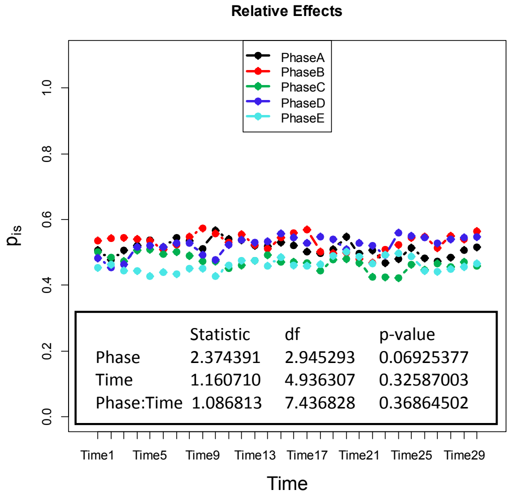

- Phase A – Baseline
- Phase B – Physiotherapy
- Phase C – WBV (whole-body-vibration)
- Phase D – Early Rest
- Phase E – Late Rest

Detailed data presentation and statistical results for longitudinal data assessment. Algorithm of Brunner used for calculation. Time indicates 30 sequential mean values (6 per min) during the compared phases.

Baseline vs. Physiotherapy

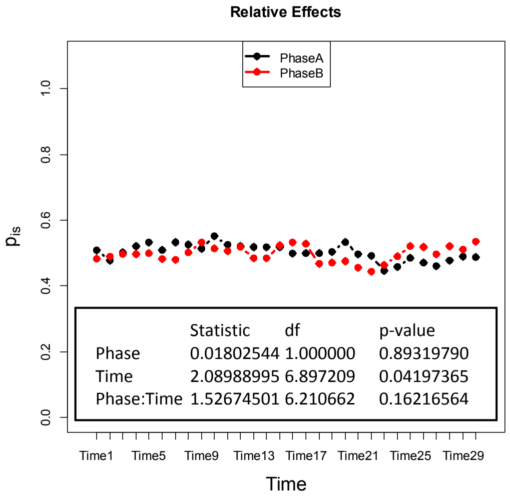

Baseline vs. Vibration

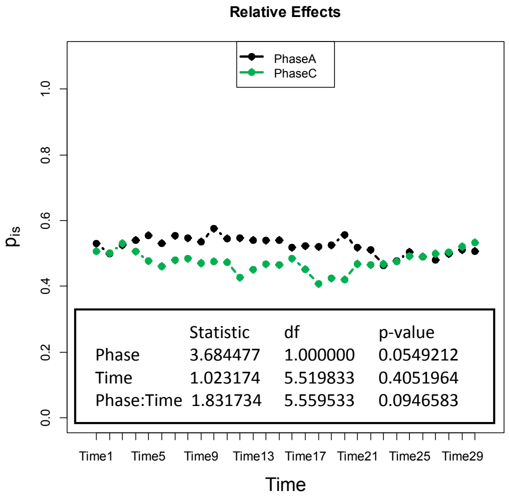

Baseline vs. Early Rest

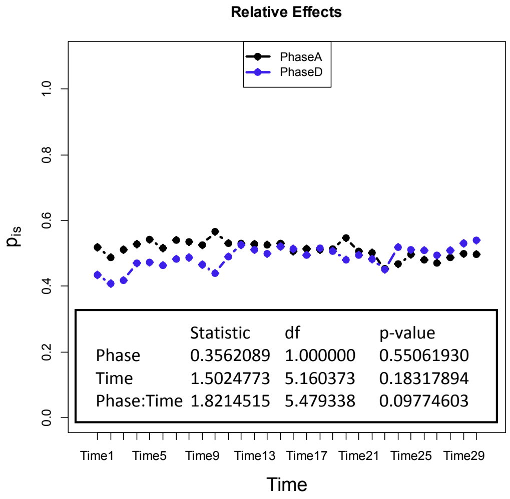

Baseline vs. Late Rest

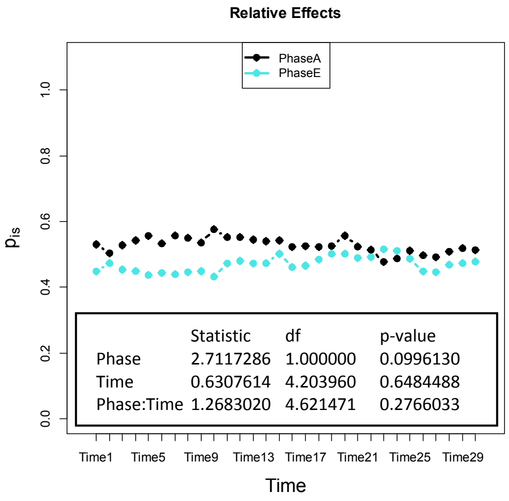

Cardiac Power Output (CPO)

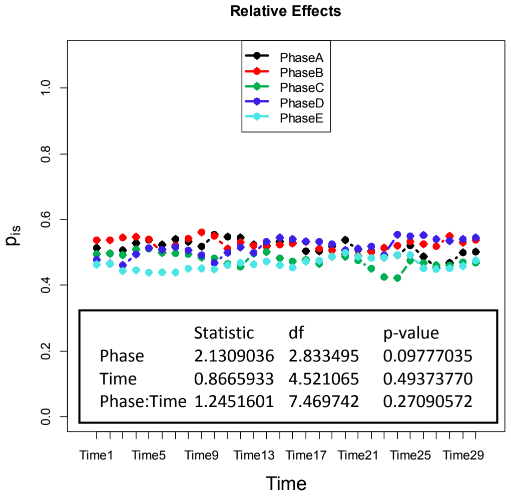

- Phase A – Baseline
- Phase B – Physiotherapy
- Phase C – WBV (whole-body-vibration)
- Phase D – Early Rest
- Phase E – Late Rest

Detailed data presentation and statistical results for longitudinal data assessment. Algorithm of Brunner used for calculation. Time indicates 30 sequential mean values (6 per min) during the compared phases.

Baseline vs. Physiotherapy

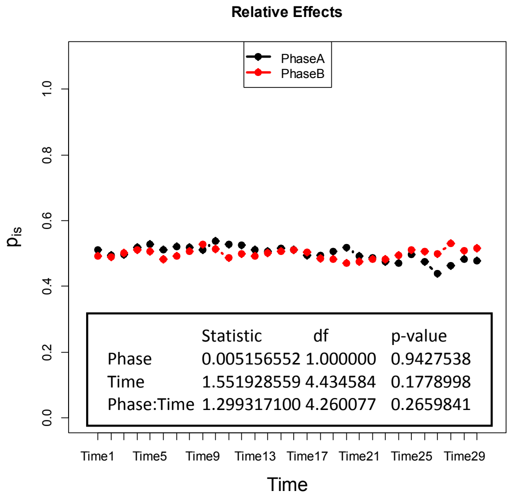

Baseline vs. Vibration

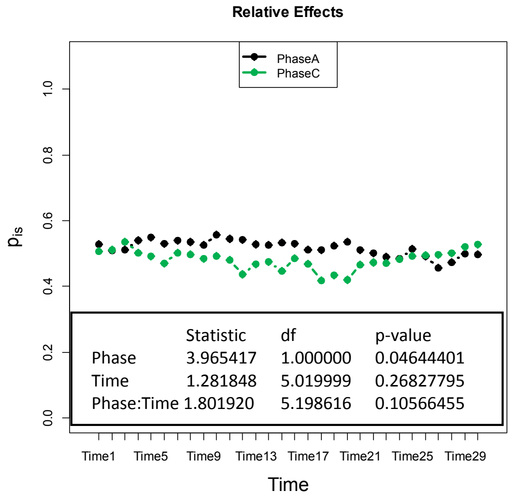

Baseline vs. Early Rest

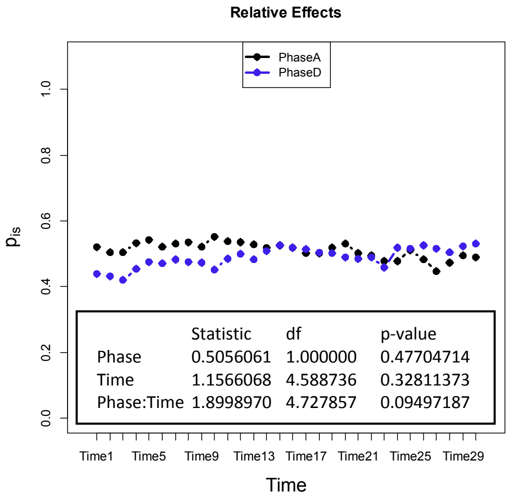

Baseline vs. Late Rest

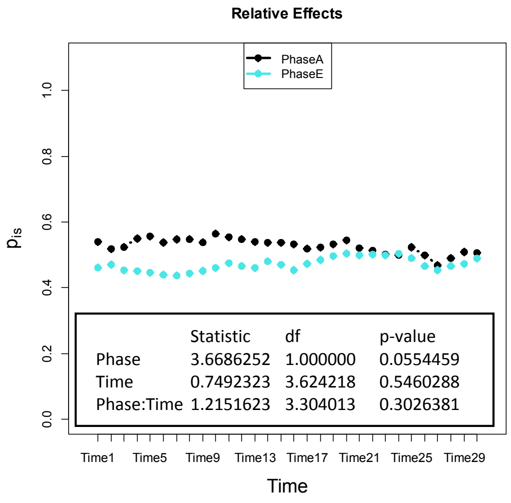

Cardiac Index (CI)

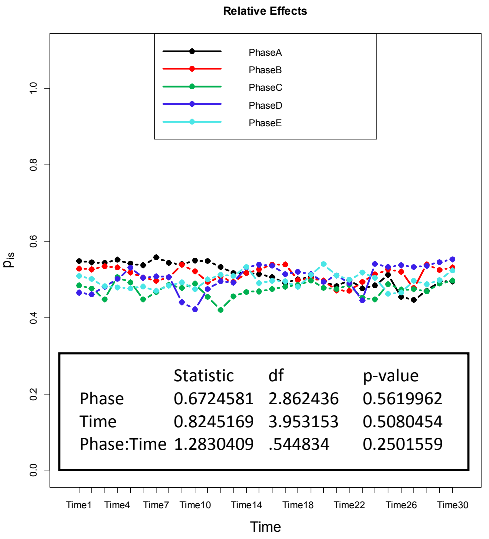

- Phase A – Baseline
- Phase B – Physiotherapy
- Phase C – WBV (whole-body-vibration)
- Phase D – Early Rest
- Phase E – Late Rest

Detailed data presentation and statistical results for longitudinal data assessment. Algorithm of Brunner used for calculation. Time indicates 30 sequential mean values (6 per min) during the compared phases.

Baseline vs. Physiotherapy

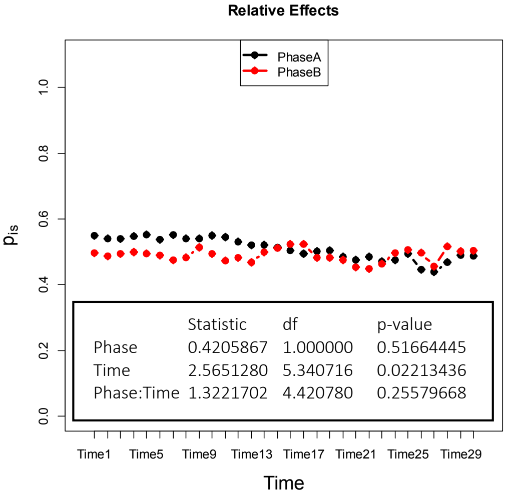

Baseline vs. Vibration

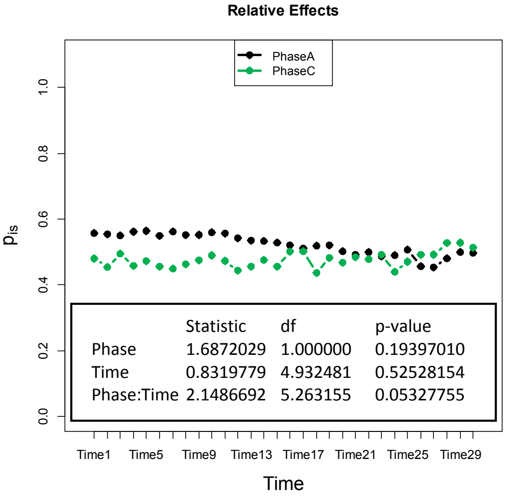

Baseline vs. Early Rest

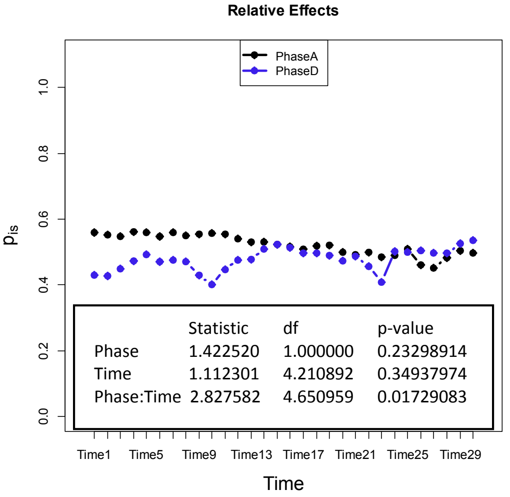

Baseline vs. Late Rest

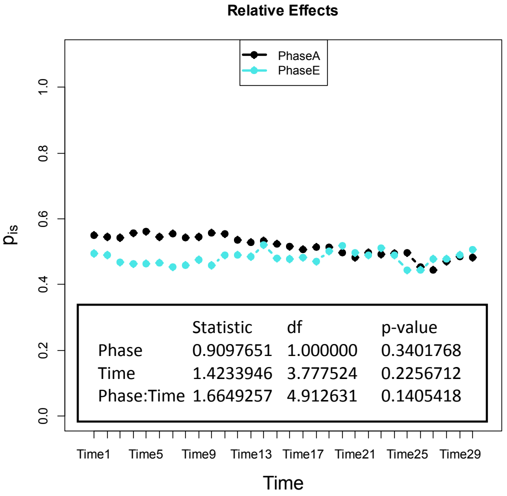

Cardiac Output (CO)

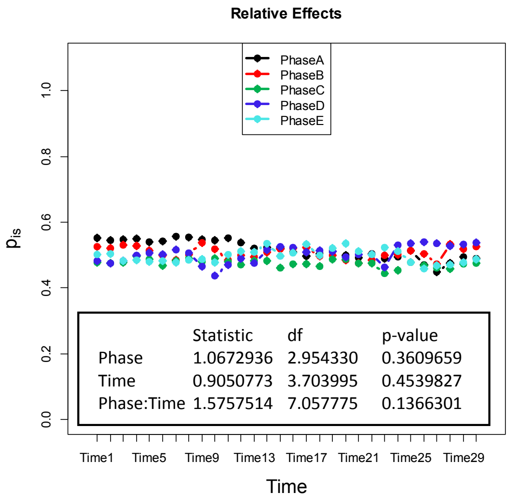

- Phase A – Baseline
- Phase B – Physiotherapy
- Phase C – WBV (whole-body-vibration)
- Phase D – Early Rest
- Phase E – Late Rest

Detailed data presentation and statistical results for longitudinal data assessment. Algorithm of Brunner used for calculation. Time indicates 30 sequential mean values (6 per min) during the compared phases.

Baseline vs. Physiotherapy

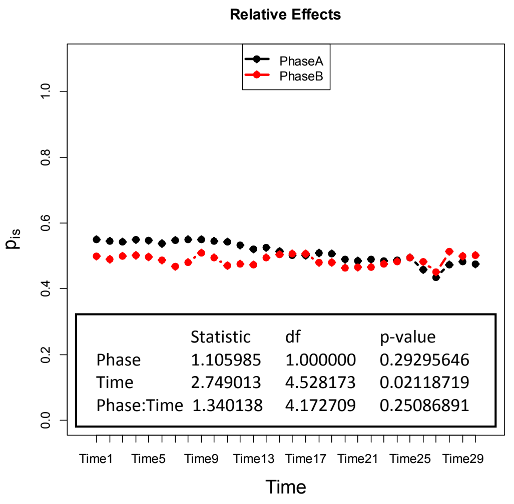

Baseline vs. Vibration

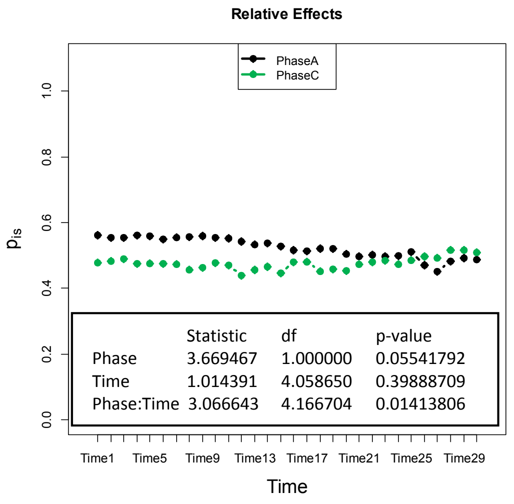

Baseline vs. Early Rest

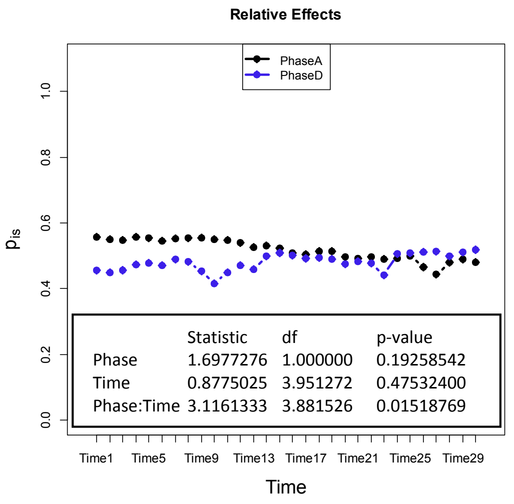

Baseline vs. Late Rest

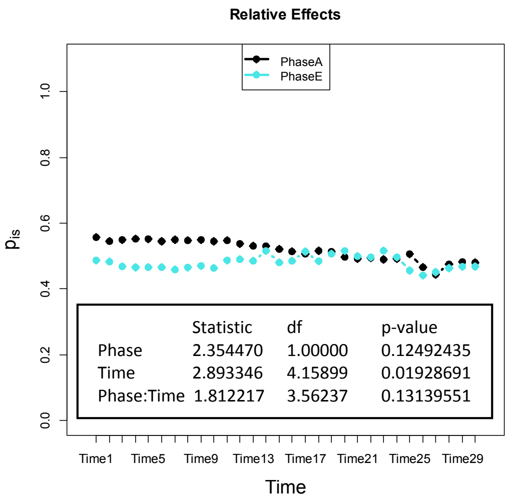

Stroke Volume (SV)

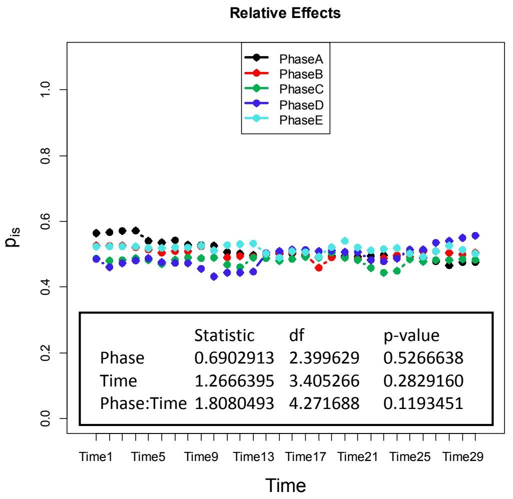

- Phase A – Baseline
- Phase B – Physiotherapy
- Phase C – WBV (whole-body-vibration)
- Phase D – Early Rest
- Phase E – Late Rest

Detailed data presentation and statistical results for longitudinal data assessment. Algorithm of Brunner used for calculation. Time indicates 30 sequential mean values (6 per min) during the compared phases.

Baseline vs. Physiotherapy

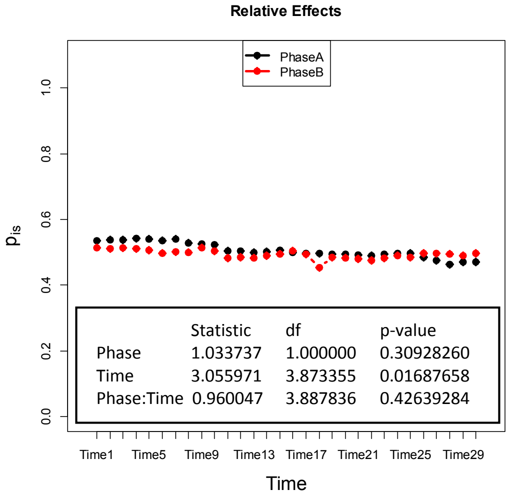

Baseline vs. Vibration

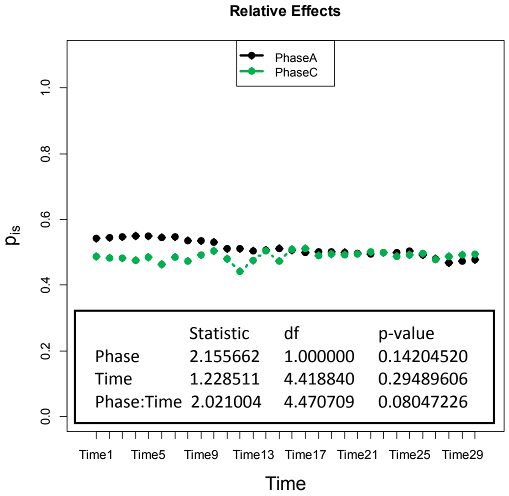

Baseline vs. Early Rest

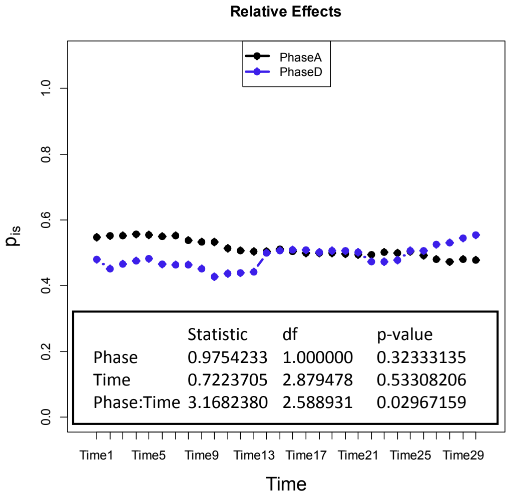

Baseline vs. Late Rest

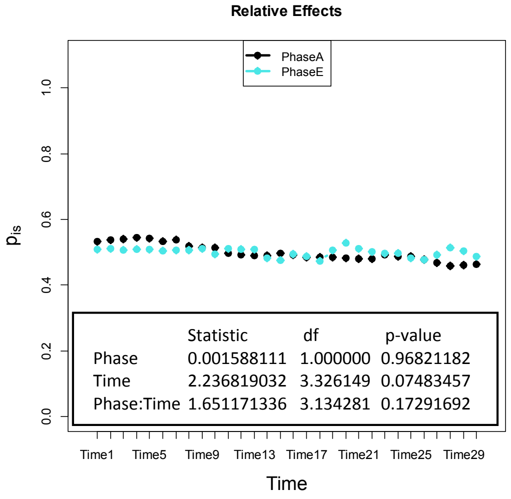

Stroke Volume Index (SVI)

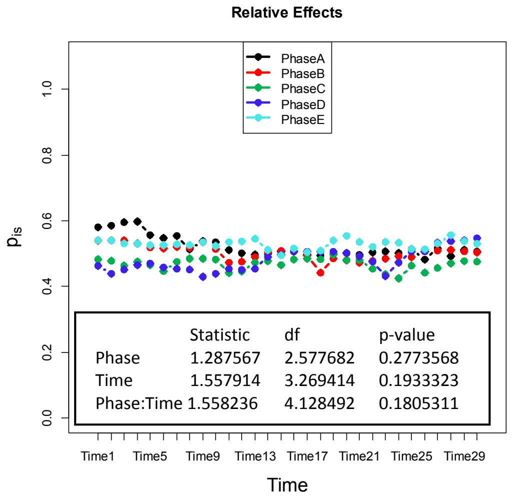

● Phase A – Baseline
● Phase B – Physiotherapy
● Phase C – WBV (whole-body-vibration)
● Phase D – Early Rest
● Phase E – Late Rest

Detailed data presentation and statistical results for longitudinal data assessment. Algorithm of Brunner used for calculation. Time indicates 30 sequential mean values (6 per min) during the compared phases.

Baseline vs. Physiotherapy

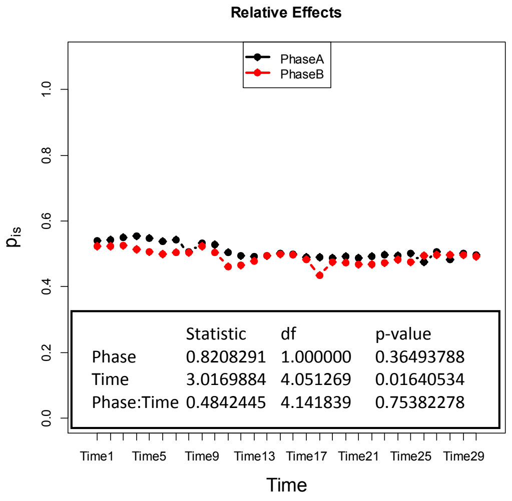

Baseline vs. Vibration

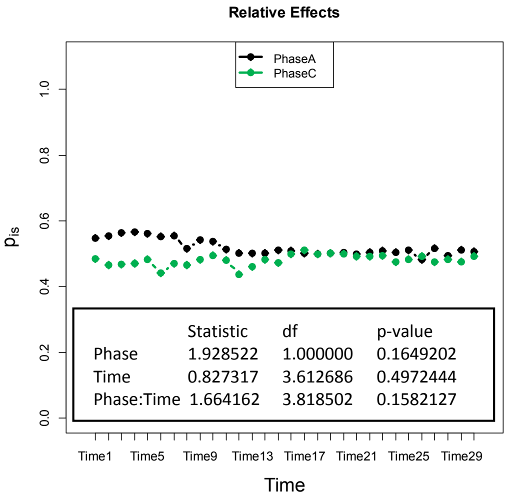

Baseline vs. Early Rest

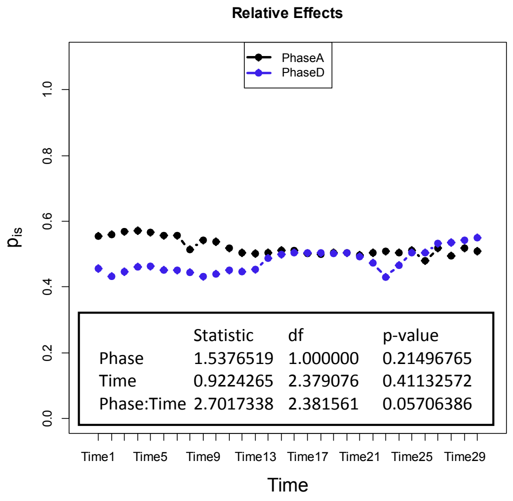

Baseline vs. late Rest

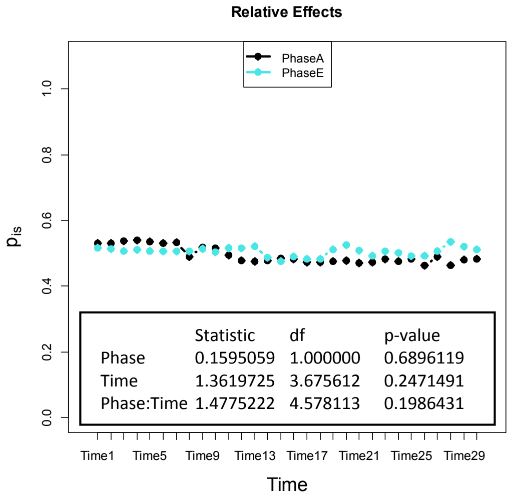

maximum Stroke Volume SVmax

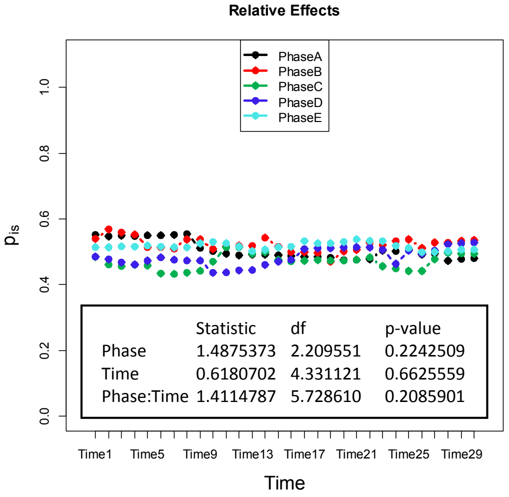

- Phase A – Baseline
- Phase B – Physiotherapy
- Phase C – WBV (whole-body-vibration)
- Phase D – Early Rest
- Phase E – Late Rest

Detailed data presentation and statistical results for longitudinal data assessment. Algorithm of Brunner used for calculation. Time indicates 30 sequential mean values (6 per min) during the compared phases.

Baseline vs. Physiotherapy

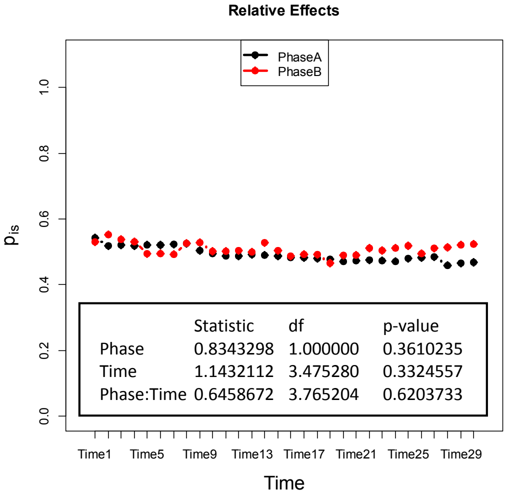

Baseline vs. Vibration

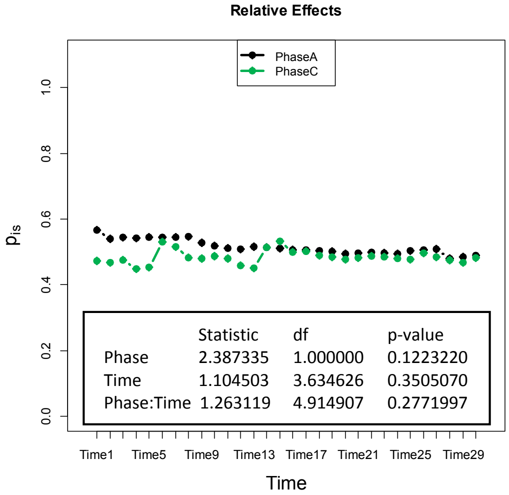

Baseline vs. Early Rest

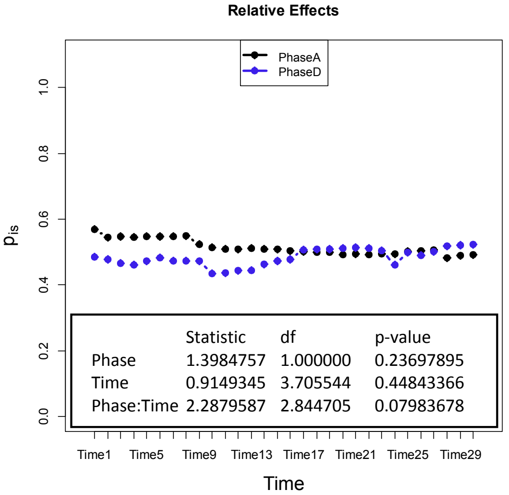

Baseline vs. Late Rest

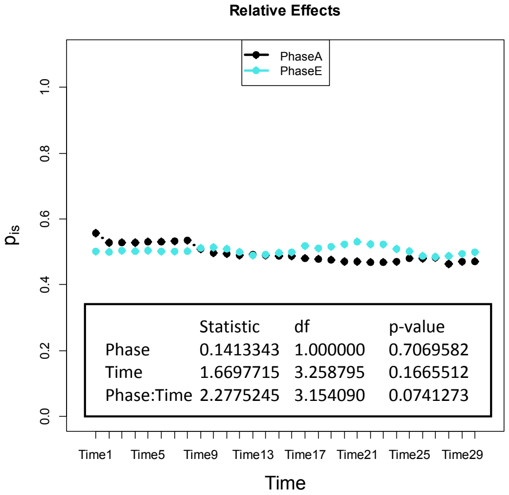

minimum Stroke Volume (SVmin)

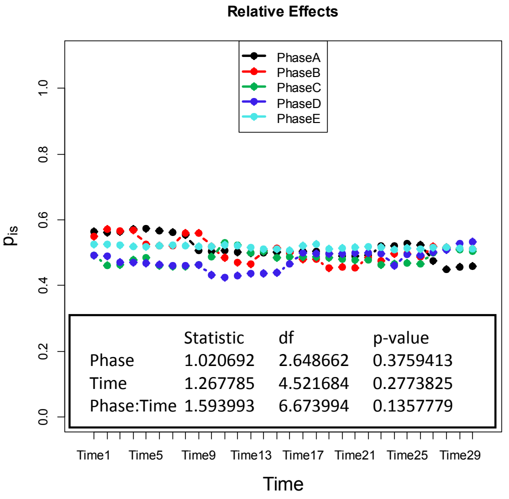

- Phase A – Baseline
- Phase B – Physiotherapy
- Phase C – WBV (whole-body-vibration)
- Phase D – Early Rest
- Phase E – Late Rest

Detailed data presentation and statistical results for longitudinal data assessment. Algorithm of Brunner used for calculation. Time indicates 30 sequential mean values (6 per min) during the compared phases.

Baseline vs. Physiotherapy

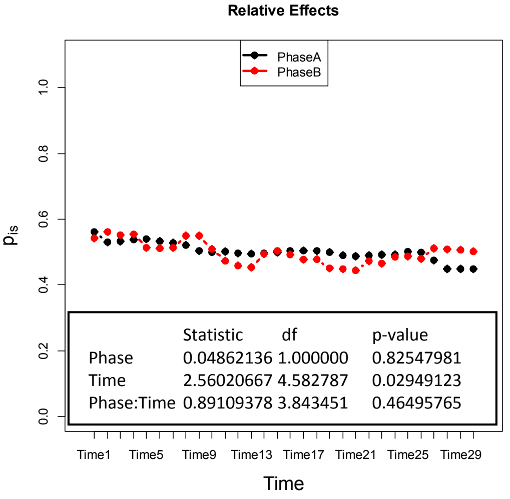

Baseline vs. Vibration

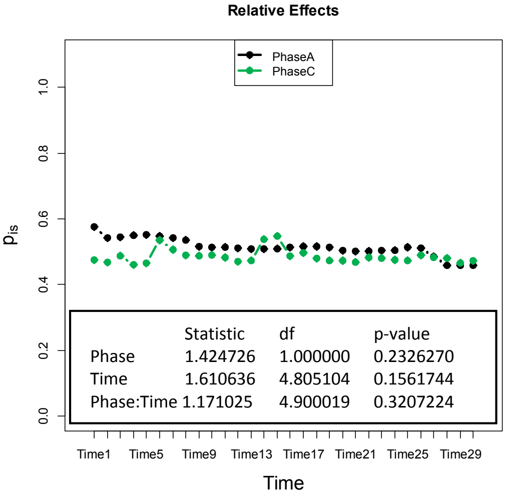

Baseline vs. Early Rest

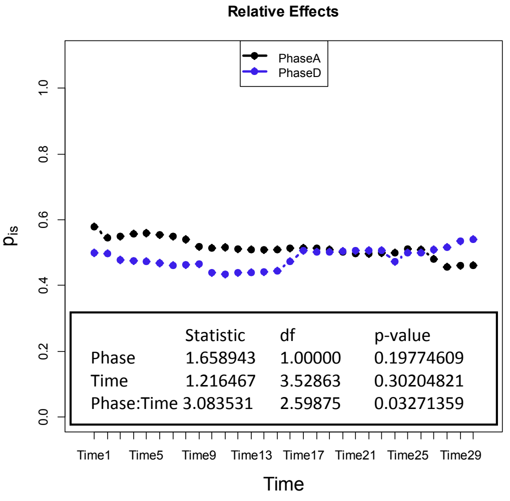

Baseline vs. Late Rest

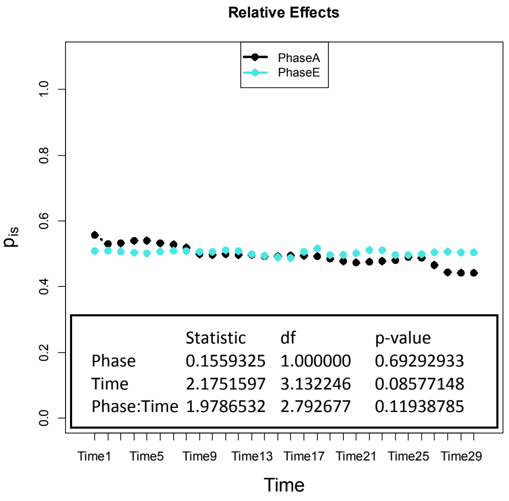

Stroke Volume Variance (SVV)

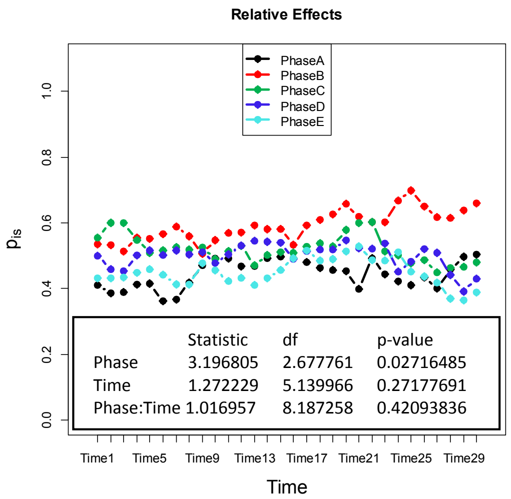

	Statistic	df	p-value
Phase	3.196805	2.677761	0.02716485
Time	1.272229	5.139966	0.27177691
Phase:Time	1.016957	8.187258	0.42093836

● Phase A – Baseline
● Phase B – Physiotherapy
● Phase C – WBV (whole-body-vibration)
● Phase D – Early Rest
● Phase E – Late Rest

Detailed data presentation and statistical results for longitudinal data assessment. Algorithm of Brunner used for calculation. Time indicates 30 sequential mean values (6 per min) during the compared phases.

Baseline vs. Physiotherapy

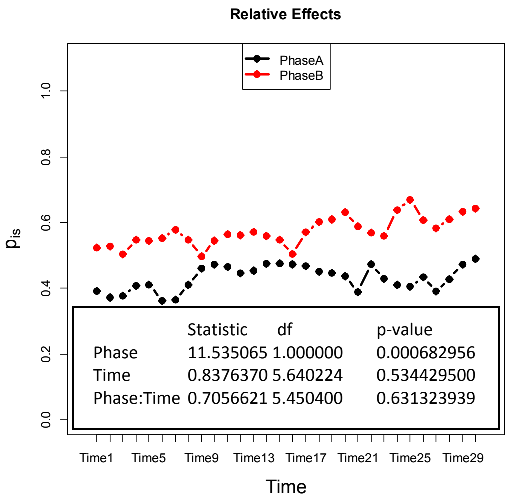

	Statistic	df	p-value
Phase	11.535065	1.000000	0.000682956
Time	0.8376370	5.640224	0.534429500
Phase:Time	0.7056621	5.450400	0.631323939

Baseline vs. Vibration

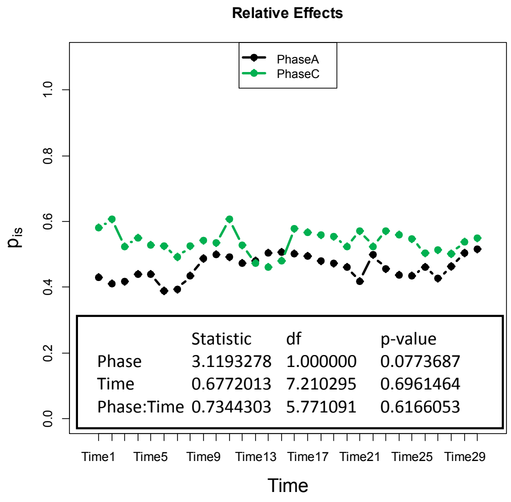

	Statistic	df	p-value
Phase	3.1193278	1.000000	0.0773687
Time	0.6772013	7.210295	0.6961464
Phase:Time	0.7344303	5.771091	0.6166053

Baseline vs. Early Rest

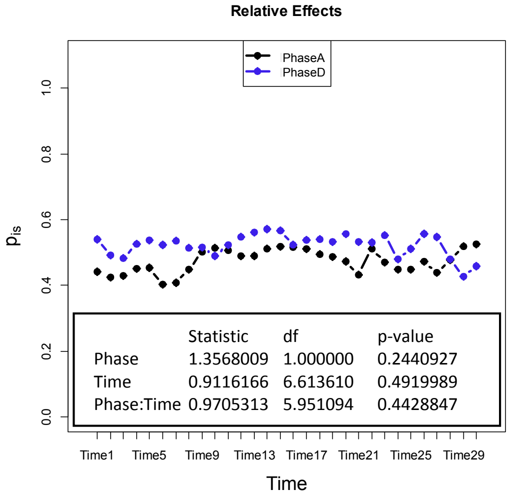

	Statistic	df	p-value
Phase	1.3568009	1.000000	0.2440927
Time	0.9116166	6.613610	0.4919989
Phase:Time	0.9705313	5.951094	0.4428847

Baseline vs. Late Rest

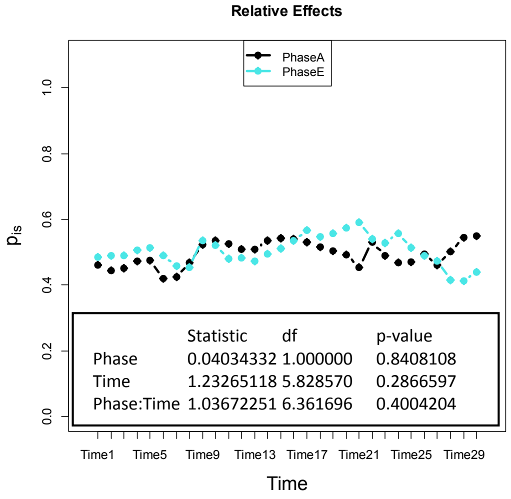

	Statistic	df	p-value
Phase	0.04034332	1.000000	0.8408108
Time	1.23265118	5.828570	0.2866597
Phase:Time	1.03672251	6.361696	0.4004204

Temperature

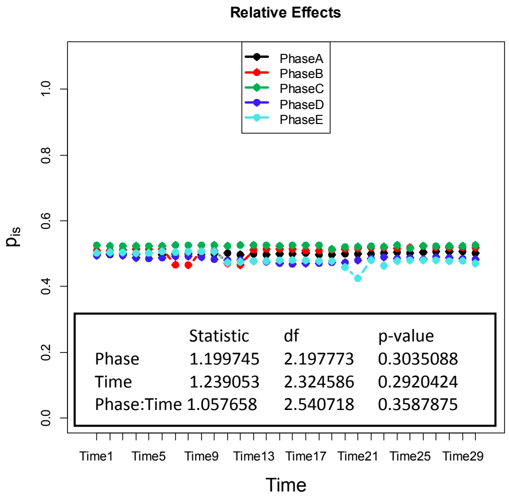

- Phase A – Baseline
- Phase B – Physiotherapy
- Phase C – WBV (whole-body-vibration)
- Phase D – Early Rest
- Phase E – Late Rest

Detailed data presentation and statistical results for longitudinal data assessment. Algorithm of Brunner used for calculation. Time indicates 30 sequential mean values (6 per min) during the compared phases.

Baseline vs. Physiotherapy

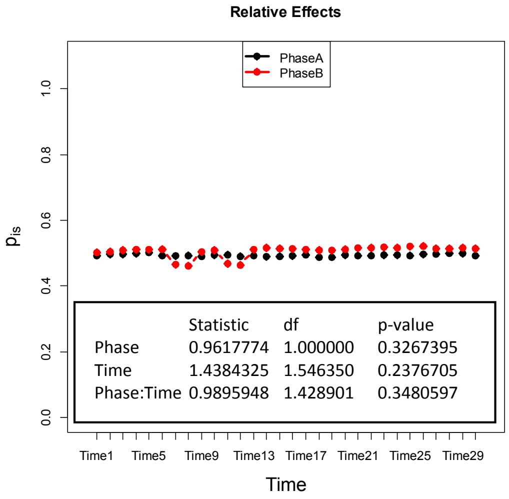

Baseline vs. Vibration

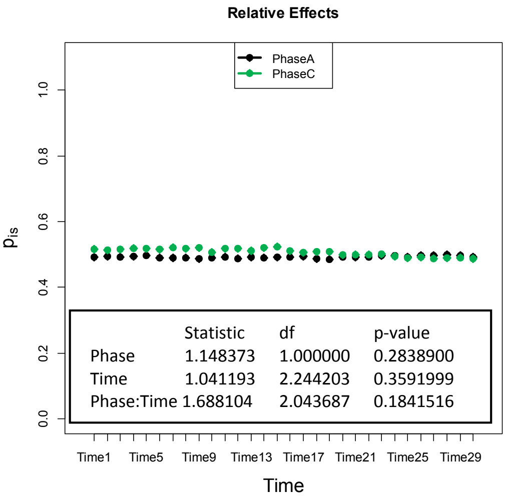

Baseline vs. Early Rest

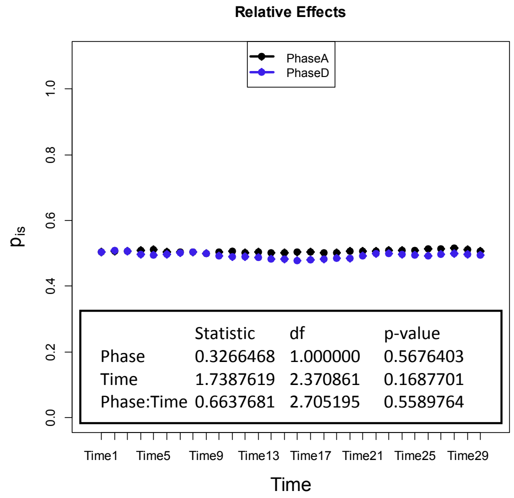

Baseline vs. Late Rest

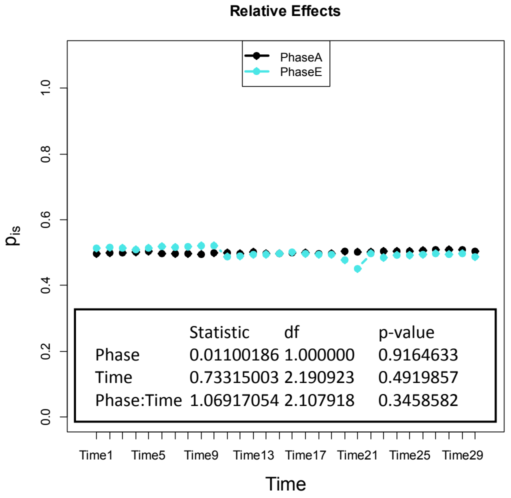

Energy metabolism

Two-factorial design (1st factor (dependent): phases, 2nd factor (dependent): Time)

<u>Respiratory Rate</u>

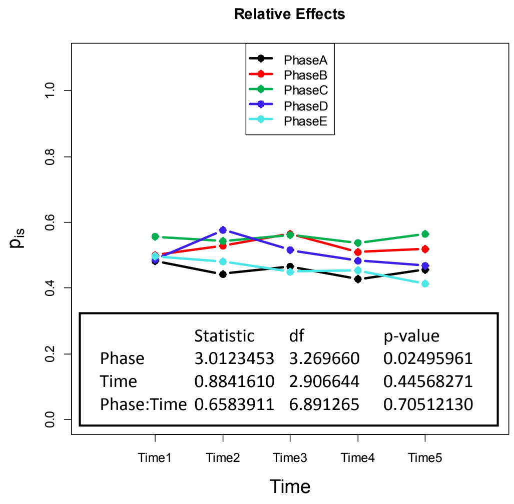

- Phase A – Baseline
- Phase B – Physiotherapy
- Phase C – WBV (whole-body-vibration)
- Phase D – Early Rest
- Phase E – Late Rest

Detailed data presentation and statistical results for longitudinal data assessment. Algorithm of Brunner used for calculation. Time indicates 5 sequential mean values (1 per min) during the compared phases.

Baseline vs. Physiotherapy

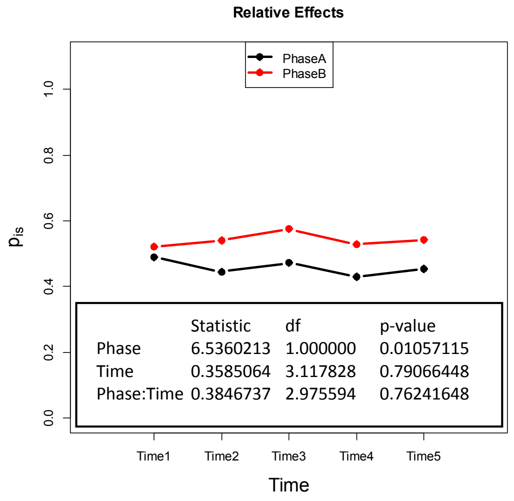

Baseline vs. Vibration

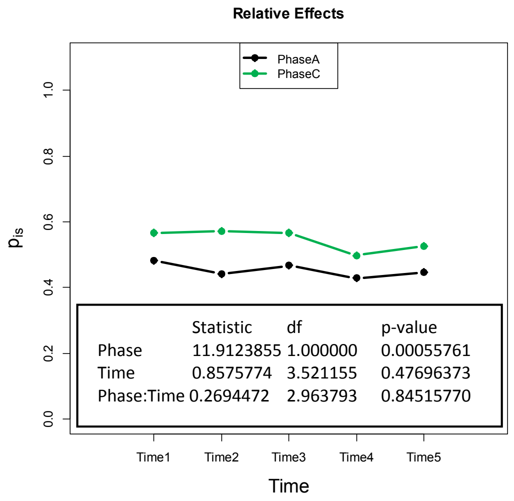

Baseline vs. Early Rest

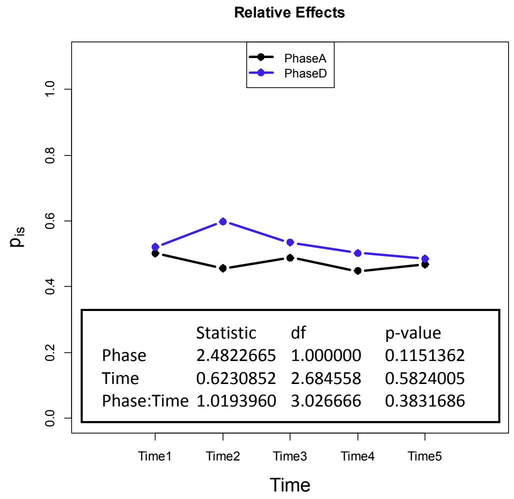

Baseline vs. Late Rest

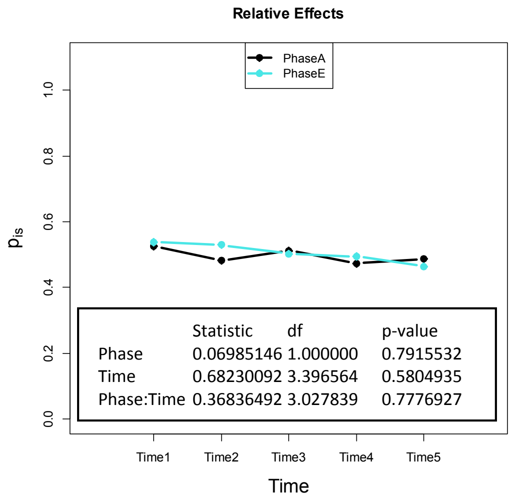

Energy Expenditure

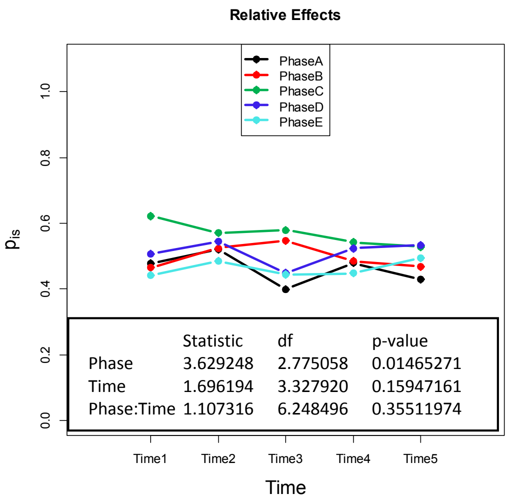

● Phase A – Baseline
● Phase B – Physiotherapy
● Phase C – WBV (whole-body-vibration)
● Phase D – Early Rest
● Phase E – Late Rest

Detailed data presentation and statistical results for longitudinal data assessment. Algorithm of Brunner used for calculation. Time indicates 5 sequential mean values (1 per min) during the compared phases.

Baseline vs. Physiotherapy

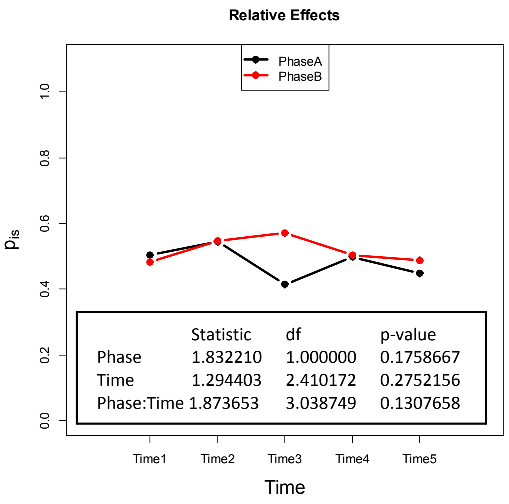

Baseline vs. Vibration

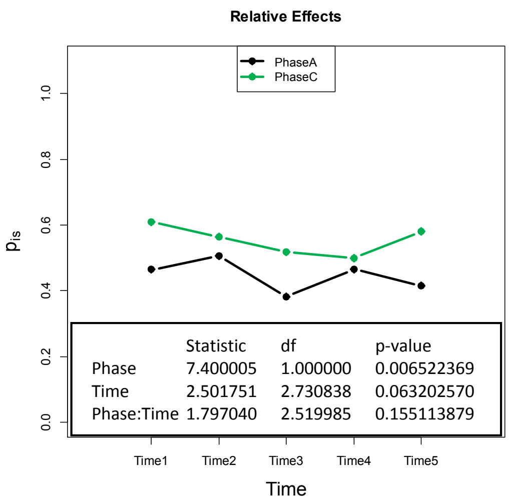

Baseline vs. Early Rest

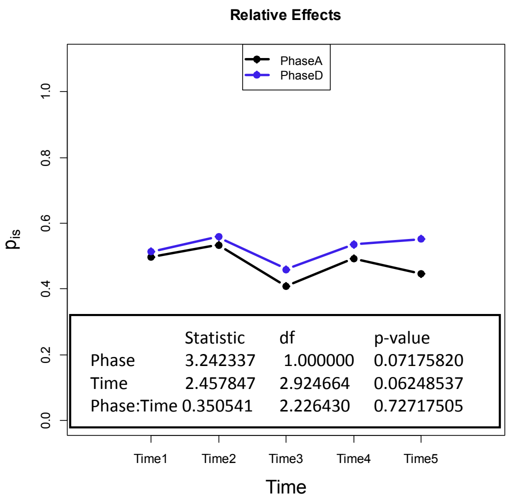

Baseline vs. Late Rest

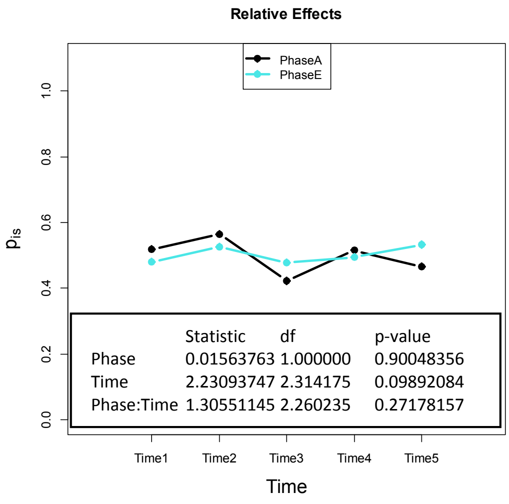

Respiratory Quotient (RQ)

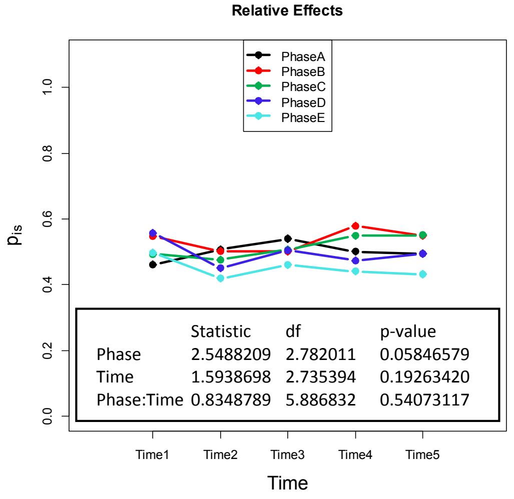

● Phase A – Baseline
● Phase B – Physiotherapy
● Phase C – WBV (whole-body-vibration)
● Phase D – Early Rest
● Phase E – Late Rest

Detailed data presentation and statistical results for longitudinal data assessment. Algorithm of Brunner used for calculation. Time indicates 5 sequential mean values (1 per min) during the compared phases.

Baseline vs. Physiotherapy

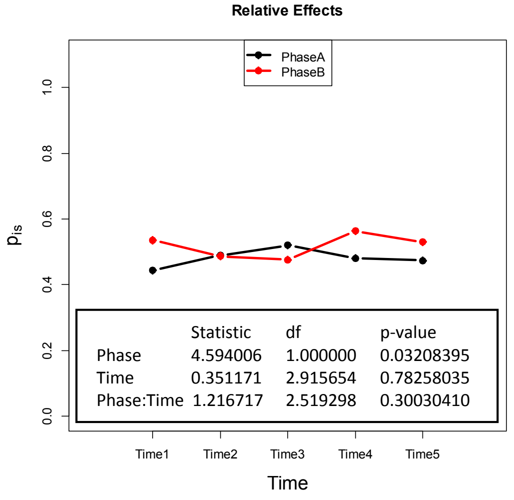

Baseline vs. Vibration

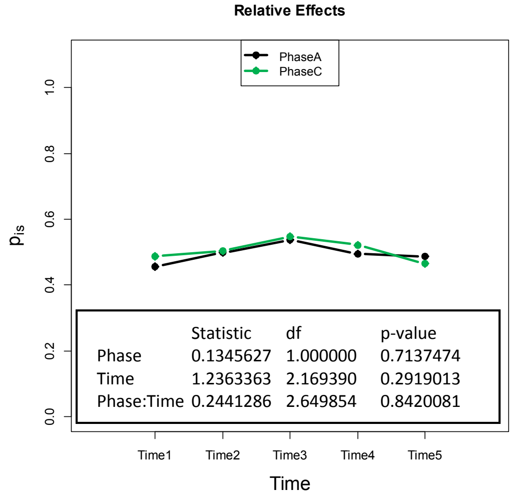

Baseline vs. Early Rest

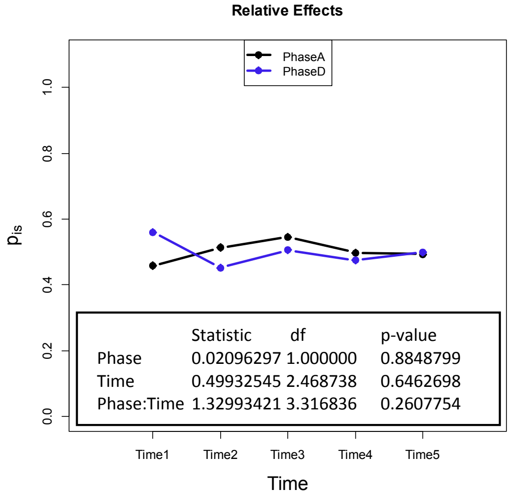

Baseline vs. Late Rest

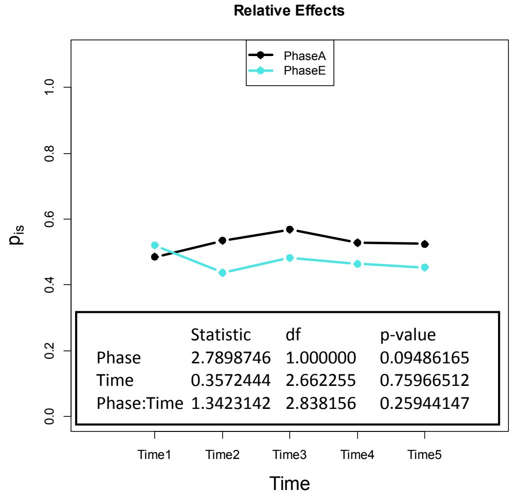

CO_2 Elimination (VCO_2)

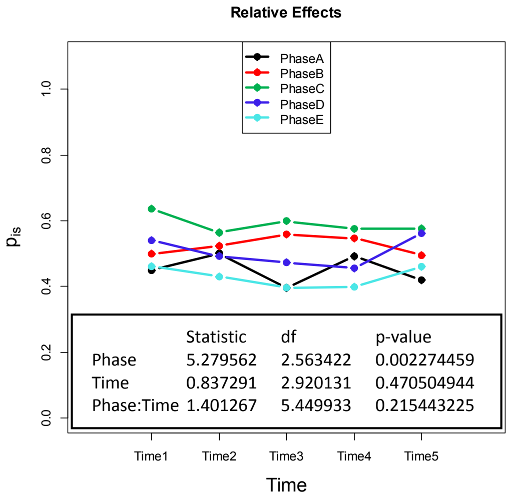

- Phase A – Baseline
- Phase B – Physiotherapy
- Phase C – WBV (whole-body-vibration)
- Phase D – Early Rest
- Phase E – Late Rest

Detailed data presentation and statistical results for longitudinal data assessment. Algorithm of Brunner used for calculation. Time indicates 5 sequential mean values (1 per min) during the compared phases.

Baseline vs. Physiotherapy

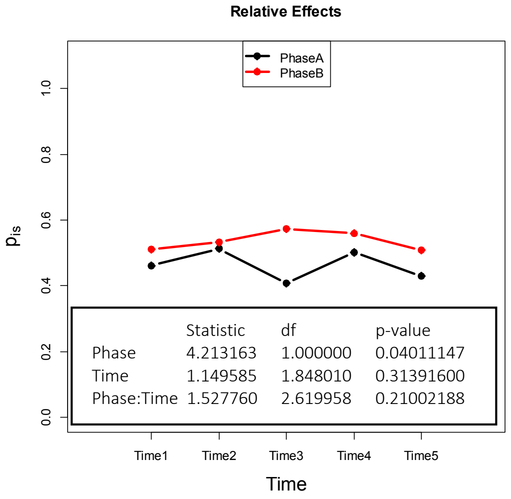

Baseline vs. Vibration

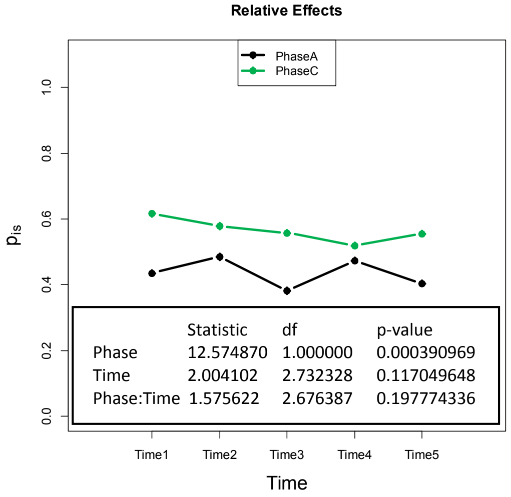

Baseline vs. Early Rest

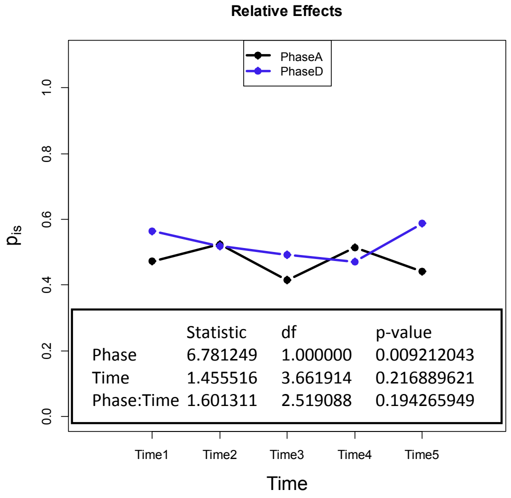

Baseline vs. Late Rest

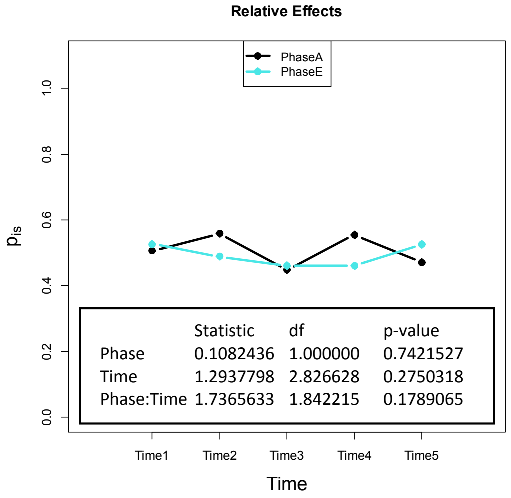

Oxygen Uptake (VO_2)

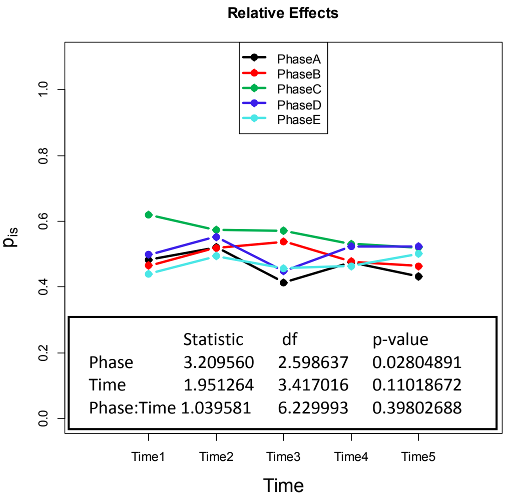

● Phase A – Baseline
● Phase B – Physiotherapy
● Phase C – WBV (whole-body-vibration)
● Phase D – Early Rest
● Phase E – Late Rest

Detailed data presentation and statistical results for longitudinal data assessment. Algorithm of Brunner used for calculation. Time indicates 5 sequential mean values (1 per min) during the compared phases.

Baseline vs. Physiotherapy

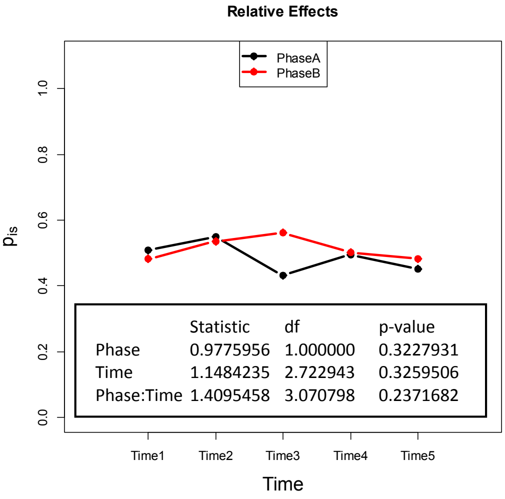

Baseline vs. Vibration

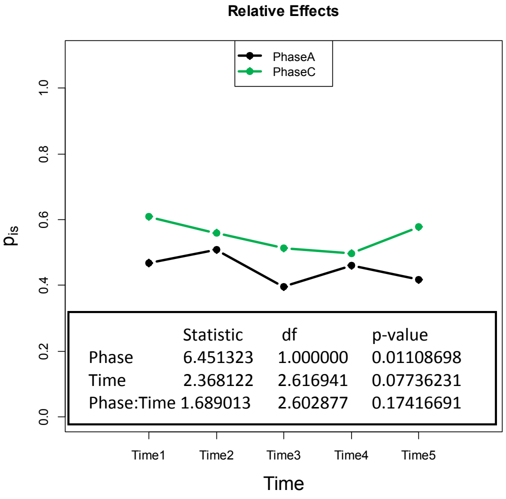

Baseline vs. Early Rest

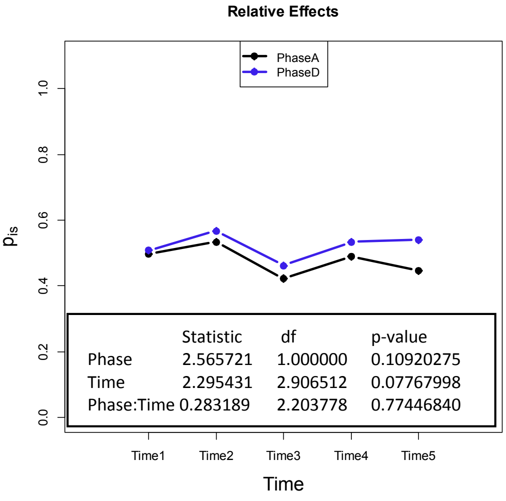

Baseline vs. Late Rest

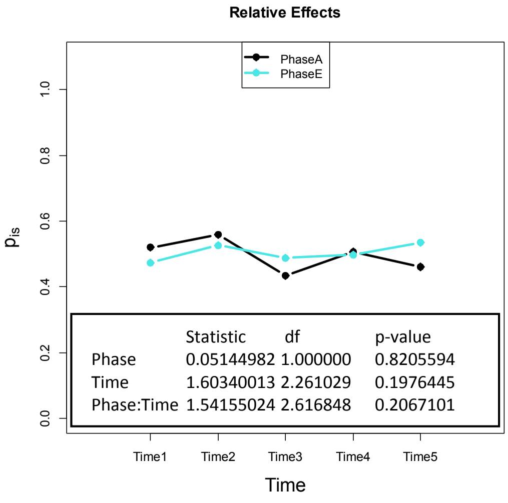

Three-factorial designs (1st factor (independent): Device, 2nd factor (dependent): Phase, 3rd factor (dependent): Time)

Table S1a Vital signs

	1st Factor (independent) Device	2nd Factor (dependent) Phase	3rd Factor (dependent) Time
**HR**	0.980	0.657	0.312
**SpO_2**	0.207	0.833	0.117
**TEMP**	0.122	0.335	0.295
**ICP**	0.923	0.134	0.371
**RR**	0.698	**0.013**	0.490

Given corresponding p-values

Table S1b Hemodynamic parameters

Parameter	1st Faktor (independent) Device	2nd Factor (dependent) Phase	3rd Factor (dependent) Time
**CI**	0.486	0.681	0.571
**MAP**	0.390	0.308	0.313
**CO**	**0.047**	0.402	0.490
**SV**	0.247	0.593	0.267
**SVI**	0.972	0.415	0.211
**SVV**	0.346	0.054	0.320
**SVMAX**	0.151	0.286	0.616
**SVMIN**	0.235	0.379	0.385
**CPI**	0.697	0.065	0.260
**CPO**	0.538	0.121	0.401
**SYSTOLE**	0.670	0.123	0.527
**DIASTOLE**	0.902	0.184	0.458

Given corresponding p-values

Table S1c Indirect calorimetry

Parameter	1st Faktor (independent) Device	2nd Factor (dependent) Phase	3rd Factor (dependent) Time
**EE**	**0.0003**	**0.024**	0.090
**RQ**	0.210	0.051	0.166
**VCO_2**	**0.002**	**0.007**	0.623
**VO_2**	**0.001**	**0.043**	0.055

Given corresponding p-values

Two-facorial designs in subgroup analyses for GAL and VIB separately

Two-facorial designs (1st factor (dependent): 2 phases each, 2nd factor (dependent): time)

Table S2a Vital signs comparing baseline and the following phases for the two devices

Parameter	VIB	GAL	BL vs. PT		BL vs. INT		BL vs. ER		BL vs. LR	
	n	n	VIB	GAL	VIB	GAL	VIB	GAL	VIB	GAL
**HR**	12	7	**0.033**	0.875	0.175	0.752	0.172	0.366	0.649	0.135
**SpO_2**	12	6	0.609	0.526	0.235	0.107	0.630	0.742	0.692	0.871
**TEMP**	10	6	**0.041**	0.394	0.341	0.235	0.717	0.817	0.587	0.992
**ICP**	5	2	0.121	0.781	0.582	0.107	0.299	0.591	0.390	**0.029**
**RR**	11	5	0.081	**0.010**	0.005	0.050	0.646	**0.008**	0.186	0.092

Vital signs comparing baseline and the following phases for the two devices, given p-values.

Table S2b Vital signs in different phases for the two devices

Parameter	Device	BL	PT	INT	ER	LR
**HR**	VIB	82.0 (17.3)	83.2 (17.5)	83.7 (17.9)	84.2 (17.6)	80.7 (19.6)
**(beats min^{-1})**	GAL	86.7 (19.5)	85.9 (18.5)	86.9 (21.2)	84.1 (18.7)	83.0 (19.6)
**SpO_2**	VIB	98.6 (2.7)	98.6 (2.6)	98.5 (2.4)	98.2 (3.0)	98.5 (3.1)
**(%)**	GAL	97.8 (2.2)	97.8 (2.9)	98.0 (2.2)	97.9 (2.0)	97.8 (2.0)
**TEMP**	VIB	37.0 (0.6)	37.0 (0.5)	37.0 (0.5)	37.0 (0.5)	37.0 (0.6)
**(Celsius)**	GAL	37.5 (0.5)	37.5 (0.6)	37.5 (0.5)	37.5 (0.6)	37.6 (0.6)
**ICP**	VIB	12.0 (5.0)	11.1 (5.4)	11.4 (3.7)	10.0 (4.3)	9.8 (5.4)
**(mmHg)**	GAL	12.0 (3.7)	10.6 (0.2)	11.4 (3.2)	12.0 (3.0)	7.9 (0.7)
**RR**	VIB	17.1 (6.2)	17.7 (6.4)	18.2 (5.5)	17.1 (6.0)	16.4 (5.4)
**(breaths min^{-1})**	GAL	16.2 (4.4)	19.6 (4.8)	19 (5.4)	18.7 (5.5)	18.8 (5.9)

Vital signs, shown in mean ± SD, of the VIB-and GAL-Groups in different phases.

Table S3a Hemodynamic parameters comparing baseline and the following phases for the two devices

	VIB	GAL	BL vs. PT		BL vs. INT		BL vs. ER		BL vs. LR	
	n	n	VIB	GAL	VIB	GAL	VIB	GAL	VIB	GAL
**CI**	9	6	0.866	0.478	0.056	0.771	0.121	0.541	0.666	0.284
**MAP**	12	7	**0.012**	0.733	0.312	0.030	0.176	0.730	0.884	0.602
**CO**	9	6	0.363	0.460	0.058	0.830	0.118	0.930	0.175	0.444
**SV**	9	6	0.055	0.458	**0.016**	0.251	**0.018**	0.373	0.888	0.737
**SVI**	9	6	0.278	0.448	**0.037**	0.458	**0.006**	0.267	0.557	0.603
**SVV**	9	6	**0.009**	0.131	0.189	0.420	0.306	0.888	0.928	0.822
**SVMAX**	9	6	0.729	0.153	**0.002**	0.168	**0.003**	0.538	0.832	0.694
**SVMIN**	9	6	**0.044**	0.526	**0.027**	0.324	**0.005**	0.606	0.753	0.954
**CPI**	9	6	0.256	0.357	0.314	0.049	0.509	0.293	0.800	**0.023**
**CPO**	9	6	0.509	0.397	0.309	0.103	0.812	0.208	0.526	**0.045**
**SYSTOLE**	12	7	0.075	0.743	0.958	**0.044**	0.287	0.910	0.850	0.490
**DIASTOLE**	12	7	**0.002**	0.433	0.130	0.372	0.068	0.959	0.724	0.919

Hemodynamic parameters comparing baseline and the following phases for the two devices, given p-values.

Table S3b Hemodynamic parameters in different phases for the two devices

Parameter	Device	BL	PT	INT	ER	LR
**CI**	VIB	3.85 (1.08)	3.85 (1.21)	3.73 (1.14)	3.75 (1.12)	3.81 (1.05)
**(L/min/m²)**	GAL	3.94 (0.54)	3.90 (0.71)	3.95 (0.60)	3.87 (0.69)	3.72 (0.56)
**MAP**	VIB	87.1 (19.2)	90.1 (17.0)	88.3 (15.2)	91.3 (17.2)	86.6 (18.6)
**(mmHg)**	GAL	83.9 (24.9)	91.8 (35.8)	79.8 (25.6)	82.7 (24.3)	85.1 (32.8)
**CO**	VIB	7.16 (2.54)	7.13 (2.80)	6.93 (2.65)	6.95 (2.59)	7.11 (2.47)
**(l/min)**	GAL	8.65 (2.66)	8.64 (3.06)	8.80 (2.69)	8.56 (2.96)	8.17 (2.56)
**SV**	VIB	87.7 (25.2)	85.4 (26.6)	82.9 (23.6)	82.4 (22.4)	88.2 (27.2)
**(ml)**	GAL	107.6 (38.0)	107.5 (43.2)	110.2 (46.4)	110.0 (43.9)	107.3 (40.8)
**SVI**	VIB	47.2 (11.5)	46.0 (12.2)	44.7 (11.1)	44.5 (10.8)	47.5 (12.7)
**(ml/min/m²)**	GAL	49.1 (12.2)	48.6 (14.1)	49.5 (16.2)	49.8 (14.5)	48.7 (13.2)
**SVV**	VIB	7.99 (6.47)	9.37 (5.03)	10.10 (6.21)	9.26 (6.70)	7.22 (5.56)
**(%)**	GAL	11.5 (8.6)	13.1 (7.4)	11.99 (7.71)	11.1 (6.5)	10.8 (6.6)
**SVMAX**	VIB	91.1 (25.8)	90.3 (28.4)	87.38 (25.51)	88.1 (26.5)	91.7 (26.9)
**(ml)**	GAL	113.3 (38.9)	113.5 (44.4)	116.37 (45.09)	115.6 (46.6)	112.4 (41.2)
**SVMIN**	VIB	83.6 (25.4)	79.7 (26.3)	79.78 (23.21)	80.9 (23.5)	85.7 (25.5)
**(ml)**	GAL	103.0 (39.0)	101.2 (41.7)	105.04 (44.62)	104.4 (41.8)	102.4 (40.5)
**CPI**	VIB	0.72 (0.25)	0.75 (0.26)	0.69 (0.22)	0.75 (0.26)	0.72 (0.22)
**(W/m²)**	GAL	0.71 (0.19)	0.68 (0.21)	0.67 (0.22)	0.67 (0.15)	0.61 (0.16)
**CPO**	VIB	1.35 (0.49)	1.39 (0.54)	1.28 (0.45)	1.38 (0.57)	1.35 (0.47)
**(W)**	GAL	1.53 (0.56)	1.48 (0.59)	1.47 (0.57)	1.44 (0.44)	1.31 (0.44)
**SYSTOLE**	VIB	139.2 (25.1)	141.0 (23.0)	138.6 (21.0)	142.5 (20.4)	138.9 (26.8)
**(mmHg)**	GAL	137.2 (28.2)	136.9 (29.5)	134.37 (29.2)	137.2 (30.4)	131.3 (27.1)
**DIASTOLE**	VIB	59.3 (13.6)	62.0 (12.7)	60.5 (11.6)	62.1 (13.2)	58.1 (13.3)
**(mmHg)**	GAL	67.5 (29.5)	67.1 (26.3)	64.5 (29.1)	65.8 (23.4)	64.8 (25.7)

Hemodynamic parameters, shown in mean ± SD, of the VIB- and GAL-Groups during different phases.

Table S4a Parameters of IC comparing baseline and the following phases for the two devices

Parameter	VIB	GAL	BL vs. PT		BL vs. INT		BL vs. ER		BL vs. LR	
	n	n	VIB	GAL	VIB	GAL	VIB	GAL	VIB	GAL
**EE**	11	5	0.515	0.291	**0.049**	0.051	0.185	0.278	0.888	0.829
**RQ**	11	5	**<0.001**	0.298	0.687	0.838	0.934	0.942	0.255	**<0.001**
**VCO_2**	11	5	**0.009**	0.333	**0.001**	**0.060**	**0.027**	0.327	0.763	0.255
**VO_2**	11	5	0.947	0.315	0.079	0.051	0.261	0.303	0.928	0.741

Parameters of IC comparing baseline and the following phases for the two devices, given p-values

Table S4b Parameters of IC in different phases for the two devices

Parameter	Device	BL	PT	INT	ER	LR
**EE (kcal per day)**	VIB	1923 (289)	1925 (332)	2079 (468)	1989 (341)	1916 (336)
	GAL	2483 (476)	2704 (574)	2800 (625)	2605 (462)	2443 (353)
**RQ**	VIB	0.74 (0.11)	0.76 (0.11)	0.75 (0.13)	0.73 (0.12)	0.72 (0.10)
	GAL	0.67	(0.08)	0.69 (0.10)	0.67 (0.10)	0.66 (0.08)
**VCO2 (ml)**	VIB	208 (29)	213 (30)	225 (42)	213 (30)	202 (25)
	GAL	251 (56)	269 (49)	284 (53)	262 (58)	243 (37)
**VO2 (ml)**	VIB	288 (47)	287 (54)	311 (75)	298 (46)	289 (55)
	GAL	376 (71)	410 (92)	423 (100)	395 (70)	372 (46)

Parameters of IC, shown in mean ± SD, of the VIB-and GAL-Groups in different phases.

Table S5a Parameters of BGA comparing baseline and the following phases for the two devices

Parameter	VIB	GAL	BL vs. INT		BL vs. ER		BL vs. LR	
	n	n	VIB	GAL	VIB	GAL	VIB	GAL
**pH**	9	6	0.789	0.091	0.349	0.310	0.146	0.398
**pCO_2**	12	7	0.666	0.075	0.307	0.345	**0.019**	1.000
**pO_2**	9	6	0.125	0.176	0.182	0.735	0.756	0.612
**HCO_3^-**	9	6	0.072	0.527	0.593	0.233	0.582	0.308
**ABE**	9	6	**0.031**	0.596	0.410	0.236	0.624	0.553
**HB**	9	6	0.116	0.611	0.346	0.340	**0.037**	**0.028**
**HCT**	9	6	0.195	0.233	0.350	0.043	0.289	0.866
**Na^+**	9	6	0.096	0.317	0.096	1.000	0.206	0.705
**K^+**	9	6	**0.020**	0.564	0.119	0.157	0.340	0.059
**GLC**	9	6	0.721	0.292	0.875	**0.028**	0.239	0.058
**LAC**	12	7	0.160	0.783	0.856	0.739	0.959	0.058

Parameters of BGA comparing baseline and the following phases for the two devices, given p-values

Table S5b Parameters of BGA in different phases for the two devices

Parameter	Device	BL	INT	ER	LR
**pH**	VIB	7.43 (0.04)	7.43 (0.04)	7.43 (0.04)	7.43 (0.03)
	GAL	7.43 (0.05)	7.44 (0.05)	7.44 (0.05)	7.44 (0.05)
**PCO_2 (mmHg)**	VIB	39.8 (3.7)	38.8 (5.8)	40.2 (3.8)	41.2 (3.1)
	GAL	40.1 (6.0)	39.1 (5.8)	39.4 (5.8)	39.9 (6.7)
**PO_2 (mmHg)**	VIB	117.6 (28.4)	108.6 (33.2)	111.6 (28.5)	115.0 (31.3)
	GAL	101.4 (39.1)	116.3 (67.1)	115.6 (77.8)	110.0 (53.8)
**HCO_3^- (mmol l^{-1})**	VIB	26.5 (2.2)	26.7 (2.0)	26.5 (2.1)	27.4 (3.6)
	GAL	26.4 (2.2)	26.5 (2.5)	26.4 (2.6)	27.7 (2.3)
**ABE (mmol l^{-1})**	VIB	2.2 (2.4)	2.5 (2.2)	2.3 (2.3)	2.3 (2.3)
	GAL	2.4 (2.3)	2.3 (2.6)	2.1 (2.7)	2.7 (2.6)
**HB (g dl^{-1})**	VIB	9.4 (0.9)	9.9 (1.0)	9.9 (1.1)	10.0 (1.1)
	GAL	9.3 (0.7)	9.7 (1.2)	9.7 (1.1)	9.9 (1.1)
**HCT (%)**	VIB	30.0 (3.1)	30.5 (3.4)	30.5 (3.5)	30.8 (3.4)
	GAL	30.0 (3.1)	29.6 (3.1)	29.3 (2.8)	29.7 (1.8)
**Na^+ (mmol l^{-1})**	VIB	142.2 (7.1)	141.8 (6.9)	141.7 (6.7)	141.8 (7.1)
	GAL	143.6 (9.2)	143.4 (6.9)	143.6 (9.2)	143.4 (9.3)
**K^+ (mmol l^{-1})**	VIB	4.2 (0.3)	4.3 (0.1)	4.3 (0.3)	4.3 (0.3)
	GAL	4.3 (0.4)	4.3 (0.3)	4.3 (0.3)	4.2 (0.4)
**GLC (mg dl^{-1})**	VIB	126.8 (24.9)	128.8 (28.0)	129.0 (28.5)	135.0 (30.9)
	GAL	134.9 (23.6)	133.1 (23.4)	127.7 (18.2)	128.1 (25.2)
**LAC (mmol l^{-1})**	VIB	11.7 (4.8)	11.3 (5.0)	11.8 (5.1)	11.7 (5.0)
	GAL	16.9 (13.2)	16.7 (14.1)	16.7 (13.8)	15.3)

Parameters of BGA, shown in mean ± SD, of the VIB-and GAL-Groups in different phases.

Abbreviations

BL = Baseline; PT = Physiotherapy; INT = Intervention; ER = Early Rest; LR = Late Rest; VIB = Vibrosphere; GAL = Galileo; CI = Cardiac index; MAP = Mean arterial pressure; CO = Cardiac output; SV = Stroke volume; SVI = Stroke volume index; SVV = Stroke volume variation; SVI = Stroke volume index; SVMAX = Maximal stroke volume; SVMIN = Minimal stroke volume; CPI = Cardiac power index; CPO = Cardiac power output; HR = Heart Rate; SpO2 = Peripheral capillary oxygen saturation; Temp = Temperature; ICP = Intracranial pressure; RR = Respiratory rate; IC = Indirect calorimetry; EE = Energy expenditure; RQ = Respiratory quotient; VCO_2 = Rate of elimination of carbon dioxide; VO_2 = Oxygen uptake; BGA = Blood gas analysis; pH = Pondus hydrogenii; pCO_2 = Partial pressure of carbon dioxide; pO_2 = Partial pressure of oxygen; HCO_3^- = Bicarbonate; ABE = Current base excess; HB = Hemoglobin; HCT = Hematocrit; Na^+ = Sodium; K^+ = Potassium; GLC = Glucose; LAC = Lactate
